# Morphological Modification of the Mouthparts of Aphids (Hemiptera: Sternorryncha: Aphididae)

**DOI:** 10.3390/insects17010087

**Published:** 2026-01-13

**Authors:** Yuchen Shi, Łukasz Depa, Jolanta Brożek, Wu Dai

**Affiliations:** 1 Key Laboratory of Plant Protection Resources and Pest Management of the Ministry of Education, College of Plant Protection, Northwest A&F University, Yangling 712100, China; yuchenshi@nwafu.edu.cn; 2 Faculty of Natural Sciences, Institute of Biology, Biotechnology and Environmental Protection, University of Silesia, Bankowa 9, 40-007 Katowice, Poland; lukasz.depa@us.edu.pl

**Keywords:** Sternorrhyncha, Aphididae, mouthparts, evolutionary adaptation

## Abstract

Comparative morphological analysis of the labrum and labium among nine aphid species—*Uroleucon* sp., *Glyphina betulae*, *Myzus cerasi*, *Panaphis juglandis*, *Chaitophorus* sp., *Lachnus roboris*, *Forda* sp., *Paracletus cimiciformis*, and *Trama* sp., belonging to six subfamilies—reveals marked interspecific variation in structure, segmentation, and sensilla equipment. The labrum is generally triangular and plate-like, ranging from 88.8 μm (*M. cerasi*) to 358.1 μm (*L. roboris*). However, *Trama* sp. exhibits a distinctive conical labrum (311.1 μm) bearing three pairs of sensilla trichodea (St2)—unique among the examined taxa. Most species possess a four-segmented labium, while *Trama* sp. and *L. roboris* exhibit five segments. The second segment is the longest and most elaborate, bearing dense arrays of sensilla or spiniform tubercles in several species. Sensilla trichodea (St1–St5) are widespread across taxa, showing the highest densities in *Chaitophorus sp* St1, *Trama* and *L. roboris* St2, *Uroleucon* sp St3, and *P. cimiciformis* St4, whereas *Trama* sp. uniquely combines sensilla St2, St3, St5, and sensilla basiconica (Sb2). Sensilla basiconica (Sb1) are consistently positioned at the base of the labrum and the fourth labial segment, except in *Trama* sp., which presents sensilla St3. Distinct cuticular modifications—including apical cuticular processes and granular protrusions—occur only in *Trama* sp. and *L. roboris*, suggesting lineage-specific adaptations. These morphological patterns indicate that aphid mouthpart diversity reflects functional specialisation linked to host-plant structural variation.

## Simple Summary

This study compares the mouthpart structures of nine aphid species, with a focus on the labrum and labium. Most species have a triangular labrum and a four-segmented labium, while *Trama* sp. and *Lachnus roboris* have five segments. *Trama* sp. stands out with a conical labrum and unique types of sensilla not found in other species. Sensilla trichodea and basiconica are distributed differently across species, with *Uroleucon* sp., *L. roboris*, and *Trama* sp. showing the highest numbers and most complex patterns. Special surface features, such as cuticular processes and granular protrusions, appear only in *Trama* sp. and *L. roboris*. These differences suggest that aphid mouthparts are adapted to different host-plant structures, reflecting their feeding specialisations and evolutionary diversification within the Aphididae.

## 1. Introduction

The piercing, sucking mouthparts of insects in the order Hemiptera represent one of their most distinctive morphological adaptations. Within the suborder Sternorrhyncha—which includes aphids, whiteflies, scale insects, and psyllids—the rostrum (labium) forms a sheath for stylets derived from the mandibles and maxillae, and its external segmentation and internal structure are critical both functionally and taxonomically [1,2]. Emeljanov [2] suggested that within Sternorrhyncha, the primitive rostrum may have been three-segmented, and that the four-segmented condition of aphids could have arisen through division of the distal part of a three-segmented rostrum.

Aphids (Hemiptera: Sternorrhyncha: Aphididae) and their close relatives in the families Adelgidae and Phylloxeridae possess highly specialised piercing–sucking mouthparts adapted to extract phloem or parenchyma sap from their host plants. The structure, segmentation, and relative length of the labium vary substantially across aphid taxa and have long been recognised as key morphological traits linked to host specificity, feeding site selection, and evolutionary adaptation [1,3]. In aphids (Aphididae), the rostrum is typically described as comprising four or five segments. Early observations by Guyton [4] noted that the “rostrum (labium) consists of four or five segments, and this segmentation is characteristic of the family.” Brożek et al. [1] corroborated this, observing that “in most aphids the rostrum is stiff and usually retracted consists of four or five segments.” Variation in the number of rostral segments, their relative lengths, and fusion of apical segments (segments IV + V called ultimate rostral segment) is correlated with feeding mode, host plant specificity, and phylogenetic lineage. For instance, in the subfamily Lachninae, the rostrum may display five discrete segments due to secondary division of the apical segment [1,5]. Also, Turpeau-Ait Ighil et al. [3] describe the rostrum as “consisting of 4 segments with unequal length (the first one can be very long),” noting that in many species the apical segments are fused or difficult to distinguish. Functionally, rostral segmentation influences the flexibility, reach, and insertion angle of the stylet bundle. Brożek et al. [1] reported in the genus *Stomaphis* Walker (Lachninae) that the first two rostral segments retract internally while only the distal segments remain externally visible during feeding, a modification associated with penetration of thick tree bark. For example, in *Stomaphis quercus* (L.) and *Stomaphis graffii* Cholodkovsky, the labium may exceed the aphid’s body length, reaching up to 13 mm. During feeding, the proximal segments retract into the body cavity, allowing the stylets to penetrate deeply into woody tissues [6]. *Cinara puerca* Hottes, which also belongs to Lachninae, was indicated to have long stylets and a long, five-segmented labium that is almost as long as the body. Like *Stomaphis*, *Cinara* telescopes the segments during feeding on pine bark [7]. By contrast, a leaf- or stem-feeding species such as *Aphis fabae* Scopoli or *Myzus persicae* (Sulzer) has a shorter, four-segmented labium that terminates near the hind coxa and is sufficient for accessing phloem near the epidermal surface [3]. Closely related Adelgidae and Phylloxeridae show analogous functional diversity. *Adelges piceae* (Ratzeburg) possesses long stylets (up to four times the body length in larvae) but a relatively short labium—an unusual configuration enabling penetration through conifer bark [8]. *Daktulosphaira vitifoliae* (Fitch), the grape *Phylloxera*, exhibits morphological differences between root-feeding and leaf-galling morphs, with the former having a noticeably longer labium, which is suited for subterranean feeding [9]. These examples collectively suggest that the labium and associated mouthpart structures evolved under selective pressures imposed by the host plant’s anatomy—especially the thickness and mechanical resistance of tissues to be penetrated. This illustrates how labium segmentation and mobility are subject to evolutionary adaptation in response to the ecological niche. Consequently, labium segmentation in aphids represents both a taxonomic character and a functional trait linked to feeding specialisation.

Many insects have diverse sensilla on their labrum and labium that have evolved to have highly specialised morphology (stiff wall of hair and flexible socket), pointing to mechanical properties in accordance with their ecological requirements [10]. These small cuticular sensory organs are crucial for detecting various cues during feeding. Various morphological types of sensilla are present on labial segments of hemipteran species [11,12,13,14] and are recognised as mechanosensory devices that detect changes in the position of the stylet fascicle and labium.

The aphid labium also harbours various mechanosensory structures that respond to deformation or pressure. These structures are believed to provide feedback on the physical interaction between the stylets and the rostral structures (labium and labrum), as well as plant tissues. It has been shown in some aphids that mechano-trichoid sensilla are primarily concentrated on the final labial segment, where they are likely to monitor stylet insertion, position, and tissue resistance [15,16].

Despite detailed ultrastructural studies of aphid mouthparts [1,17], the morphology, distribution, and functional role of mechanosensilla on the labium remain relatively underexplored. Mechanosensory input is particularly important in aphids, as feeding involves navigating intercellular spaces, piercing cell walls, avoiding damage from the stylet, and selecting suitable sites within the sieve tubes. The labium guides the stylet bundle and frequently telescopes or folds during insertion, suggesting that sensilla provide critical feedback about labial deformation and stylet position [1].

In other sternorrhynchan insects, the labial tip commonly bears a mixture of mechanosensilla and chemosensilla arranged in sensory fields [18,19]. In aphids, mechanosensilla such as trichoid or chaetic sensilla are often located along the margin of the labial tip and the sides of the stylet groove, presumably detecting stylet motion relative to the labium or contact with the plant surface [16]. The density and arrangement of accessory setae on the ultimate rostral segments have been used in aphid taxonomy, highlighting the sensory significance of these regions [20].

Mechanosensilla on the aphid labium likely fulfil multiple functions: monitoring resistance encountered by stylets during penetration, guiding proper tissue selection [21], informing labial segment positions during telescoping [1], and preventing stylet damage [16,22]. Nevertheless, detailed ultrastructural data, quantitative analyses of mechanosensilla distribution, interspecific comparisons, and functional studies (e.g., electrophysiology or behavioural assays) remain limited. Addressing these gaps will provide critical insights into the mechanosensory basis of aphid feeding behaviour and its evolutionary and ecological significance.

Despite numerous case studies, a comprehensive understanding of rostral morphology modification (labrum and labium) and sensilla diversity across aphid lineages remains lacking.

This study presents our original quantitative and qualitative data on rostrum structure, sensilla morphology, and distribution in representative Aphididae taxa. We test the hypothesis that the diversification of rostrum and sensilla traits in aphids is associated with host-plant niche differentiation (leaf, stem, trunk, or root feeding), and we discuss the evolutionary trends underlying these adaptations.

## 2. Materials and Methods

### 2.1. Samples in SEM

Materials for the study of aphid species representing different feeding sites on host plants were collected by the Zoological Team of the University of Silesia in Katowice, Poland. These species have been preserved in 70% ethanol. The materials of nine adult specimens were dehydrated in a series of ethanol (70–100%) and then dried according to the general procedure in a critical point dryer (CPD 300, Leica Microsystems, Wetzlar, Germany). The basal part of the head of nine species of aphids, with a part of the rostrum (labium and labrum) or the whole specimen, was glued onto a scanning electron microscope stub. The materials used for SEM photographs were gold-coated with a layer of gold (30 nm) using a Q150T ES sputter coater (Quorum Technologies Ltd., Laughton, UK). SEM micrographs were obtained using a Phenom XL (Phenom-World B.V., Eindhoven, The Netherlands) at 15 kV accelerating voltage, with a Back Scatter Detector (BSD), a Hitachi UHR FE-SEMSU8010 field emission scanning electron microscope (Hitachi High Technologies Corporation, Tokyo, Japan) with a secondary-electron detector (ESD) at 10 kV accelerating voltage, and a scanning electron microscope (SEM-FIB) (TESCAN Amber X) (Brno, Czech Republic).

### 2.2. Collection, Voucher Materials, and Descriptions of the Specimens

In this study, eight morphs of apterous viviparous females were used, and only *Panaphis juglandis* was an alate viviparous female. All species determinations were performed following key by Blackman and Eastop [23]. Collection data of the studied specimens with accession numbers of the voucher material deposited in the entomological collection of the University of Silesia in Katowice (DZUS):

*Uroleucon* sp. coll. 20.06.2025. on *Cichorium intybus*, in Krośnice, Poland, leg. Ł. Depa; feeding on stems and bases of inflorescenses; not visited by ants; det. Ł. Depa; apterous viviparous females; voucher material-microscopic slide no. DZUS 20.06.25.14-25.

*Glyphina betulae* coll. 20.06.2025. on *Betula pendula*, in Krośnice, Poland, leg. Ł. Depa; green tips of young branches with young leaves, not attended by ants. det. Ł. Depa; apterous viviparous females; voucher material-microscopic slide no. DZUS 20.06.25.15-25.

*Myzus cerasi* coll. 20.06.2025. on *Prunus avium*, in Krośnice, Poland, leg. Ł. Depa; in young, curled leaves, in very abundant colonies; not visited by ants; det. Ł. Depa; apterous viviparous females; voucher material-microscopic slide no. DZUS 20.06.25.16-25.

*Panaphis juglandis* coll. 23.06.2025. on *Juglans regia*, in Krośnice, Poland, leg. Ł. Depa; on upper surface of leaves, along main veins; visited by *Lasius niger*; det. Ł. Depa; alate viviparous females; voucher material-microscopic slide no. DZUS 23.06.25.25-25.

*Chaitophorus* sp. coll. 23.06.2025. on *Populus tremulae*, in Krośnice, Poland, leg. Ł. Depa; on undersides of young leaves; visited by *Formica cinerea*; apterous viviparous females; voucher material-microscopic slide no. DZUS 23.06.25.31-25.

*Lachnus roboris* coll. 26.06.2025. on *Quercus robur*, in Krośnice, Poland, leg. Ł. Depa; on 2–3 years old, woody branches; visited by *Lasius niger*; apterous viviparous females; voucher material-microscopic slide no. DZUS 26.06.25.38-25.

*Forda* sp. coll. 02.08.2025. on *Bromus carinatus*, in Piekary Śląskie, Poland, leg. Ł. Depa; on roots; visited by *Lasius niger*; apterous viviparous females; voucher material-microscopic slide no. DZUS 2.08.25.72-25.

*Paracletus cimiciformis* coll. 27.07.2025 on *Poa compressa*, in Rybnik, Poland, leg. Ł. Depa; few specimens were feeding on roots, but most were loosely walking in ant chambers among ant brood; in colony of *Tetramorium* cf. *caespitum*. apterous viviparous females; voucher material-microscopic slide no. DZUS 27.07.25.67-25.

*Trama* sp. coll. 24.08.2025. on *Artemisia campestris*, in Kołobrzeg, Poland, leg. Ł. Depa; on roots and underground rhizomes; visited by *Lasius niger*; apterous viviparous females; voucher material-microscopic slide no. DZUS 24.08.25.90-25

### 2.3. Position of the Sensilla and Sample Size

**Figure 1:**
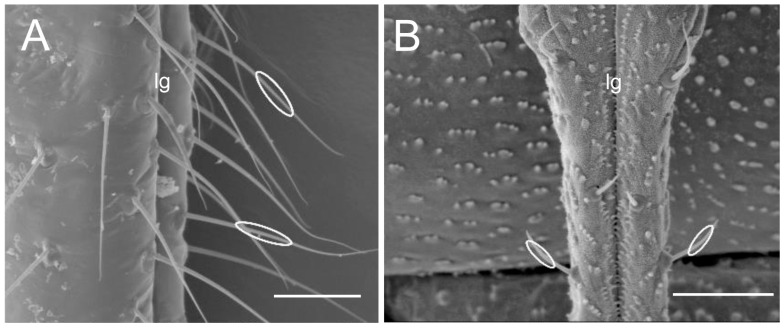
The positions for measuring the sensilla on the surface of the labium. (**A**) Example of the selection and measurement of the length of sensilla trichodea St1. (**B**) An example of the selection and measurement of the length of sensilla trichodea St3. Bars = 30 µm. Abbreviation: lg, labial groove.

Five specimens of each species were selected for the recognition of particular types of sensilla. The position for measuring the sensilla on the surface labrum and labium, and apex of the labium, was selected in the same way for all specimens, with the sensilla visible laterally to the surface (see Figure 1). The labium was positioned as parallel as possible to the field of view, and sensilla were found in a natural position, not destabilised by electrons in a scanning electron microscope. The lateral position of the sensilla shows their true length from the socket to the distal end.

Sample size: Eight sensilla were measured from each specimen for sensilla types St1–4 and Sb2–3. Five sensilla were measured for sensilla of particular types, St5, Sb1, and Sb4–5; in *Trama* sp., five sensilla were measured for sensilla type St3.

The morphological identification of the sensilla and the analysis of their features conducted in the present study were based on Altner and Prillinger [24] and Shields [25].

## 3. Results

**Figure 2:**
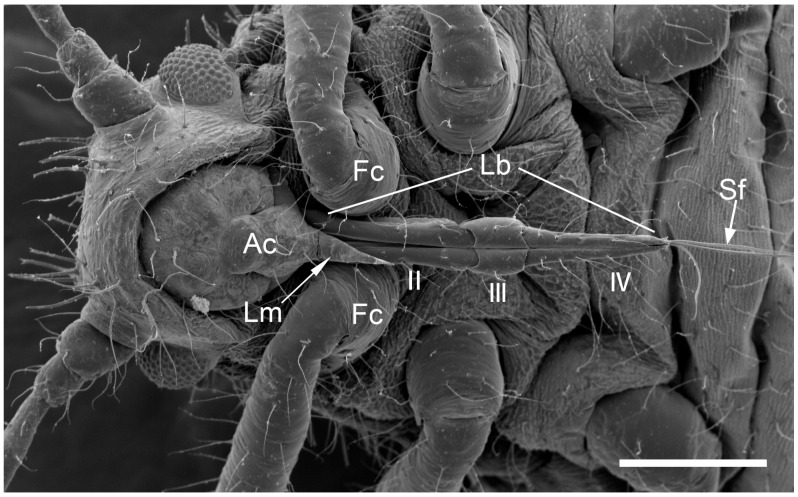
Ventral view of *Chaitophorus* sp. showing the mouthparts extended from between the forecoxae (Fc). Abbreviations: II–IV, different labium segments; Ac, anteclypeus; Lm, laburm; Lb, Labium; Sf, stylet fascicle. Bars = 200 μm.

**Figure 3:**
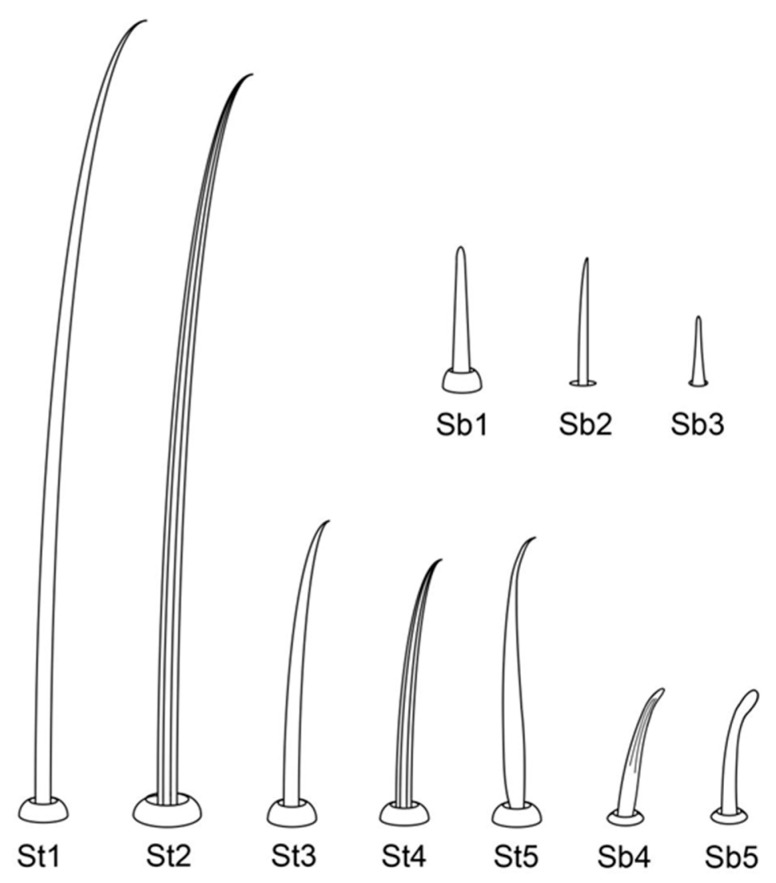
Schema of the base morphology of two types of sensilla with several subtypes on the labium and labrum in studied aphids: St1–St5, sensilla trichodea; Sb1–Sb5, sensilla basiconica.

**Figure 4:**
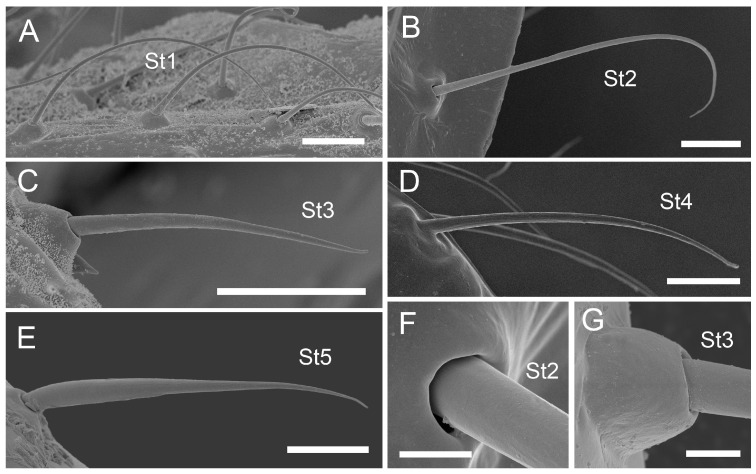
Morphology of the sensilla trichodea (St1–St5): (**A**) Sensilla St1 in *Panaphis juglandis*. (**B**) Sensilla St2 in *Lachnus roboris*. (**C**) Sensilla St3 in *Myzus cerasi*. (**D**) Sensilla St4 in *Paracletus cimiciformis*. (**E**). Sensilla St5 in *Trama* sp. (**F**,**G**) Enlarged view of the sensilla base showing flexible socket. Bars: (**A**–**E**) = 10 μm; (**F**,**G**) = 2 μm.

**Figure 5:**
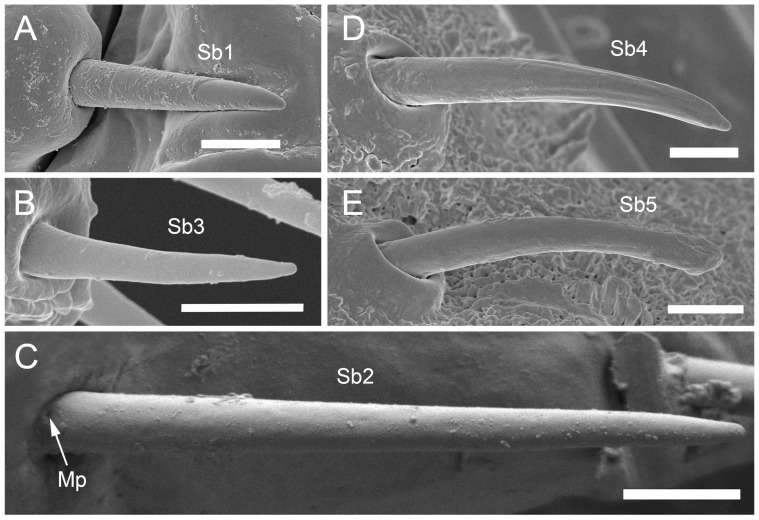
Morphology of the sensilla basiconica (Sb1–Sb5). (**A**) Sensilla Sb1 in *M. cerasi*. (**B**) Sensilla Sb3 in *Chaitophorus* sp. (**C**) Sensilla Sb2 in *Trama* sp. (**D**,**E**) Sensilla Sb4 and Sb5 in *Forda* sp. Abbreviations: Mp, moulting pore; bars, (**A**–**E**) = 2 μm.

**Figure 6:**
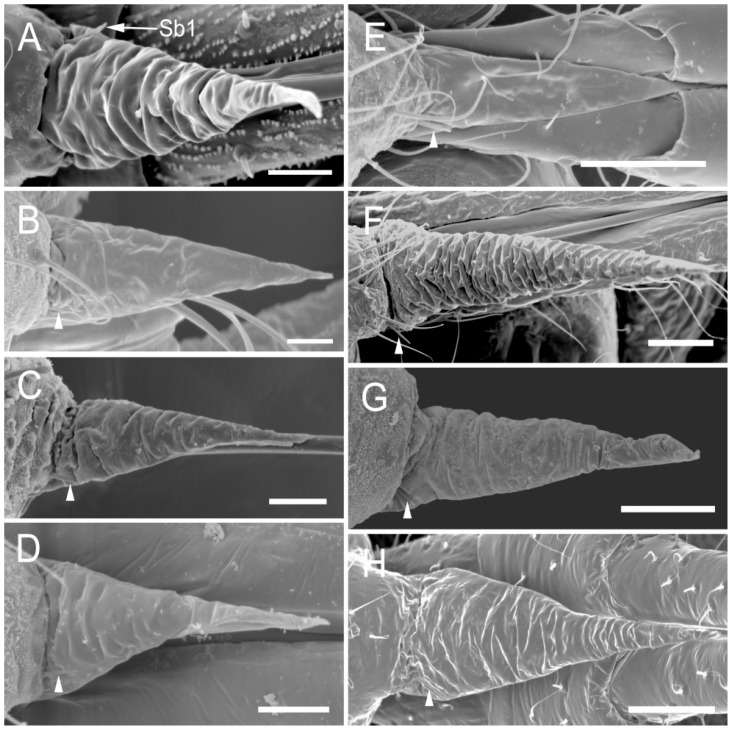
Shape of the labrum from ventral view. (**A**) *Uroleucon* sp. (**B**) *G. betulae*. (**C**). *M. cerasi*. (**D**). *P. juglandis*. (**E**) *Chaitophorus* sp. (**F**) *L. roboris*. (**G**) *Forda* sp. (**H**) *P. cimiciformis*. Abbreviations: white triangle shows the location of sensilla basiconica 1 (Sb1); bars: (**A**–**D**) = 20 μm; (**E**–**H**) = 50 μm.

**Figure 7:**
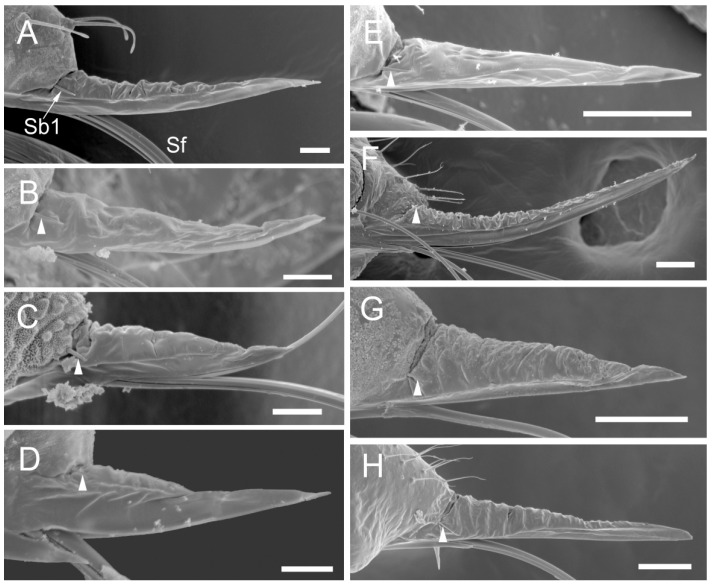
Shape of the labrum from lateral view. (**A**) *Uroleucon* sp. (**B**) *G. betulae* (**C**) *M. cerasi*. (**D**) *P. juglandis*. (**E**) *Chaitophorus* sp. (**F**) *L. roboris*. (**G**) *Forda* sp. (**H**) *P. cimiciformis*. Abbreviations: white triangle shows the location of sensilla Sb1; Sf, stylet fascicle; bars: (**A**–**D**) = 20 μm; (**E**–**H**) = 50 μm.

**Figure 8:**
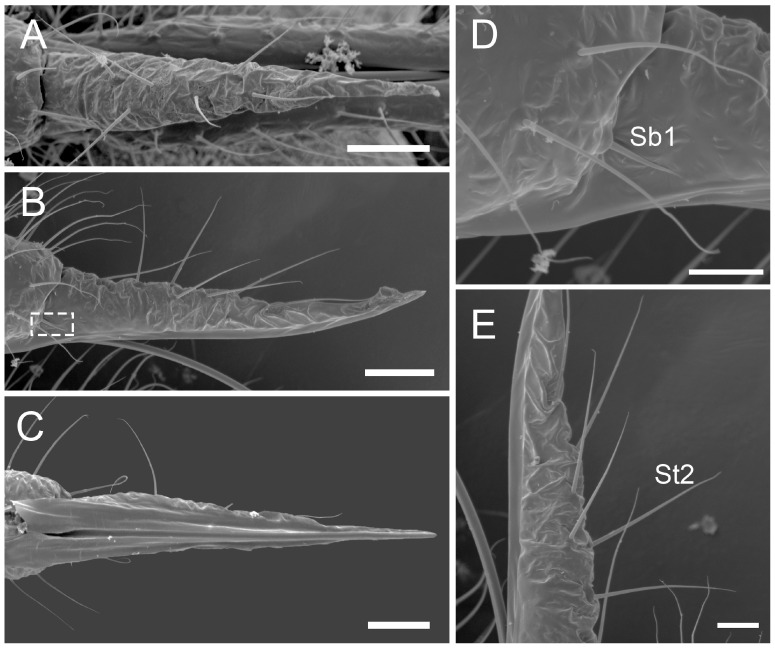
Shape of the labrum of *Trama* sp. (**A**) Ventral view. (**B**). Lateral view. (**C**) Dorsal view. (**D**) Enlarged view of sensilla Sb1 (Sb1) in (**B**) (white dotted box). (**E**) Enlarged view of sensilla St2. Bars, (**A**–**C**) = 50 μm; (**D**,**E**) = 20 μm.

**Figure 9:**
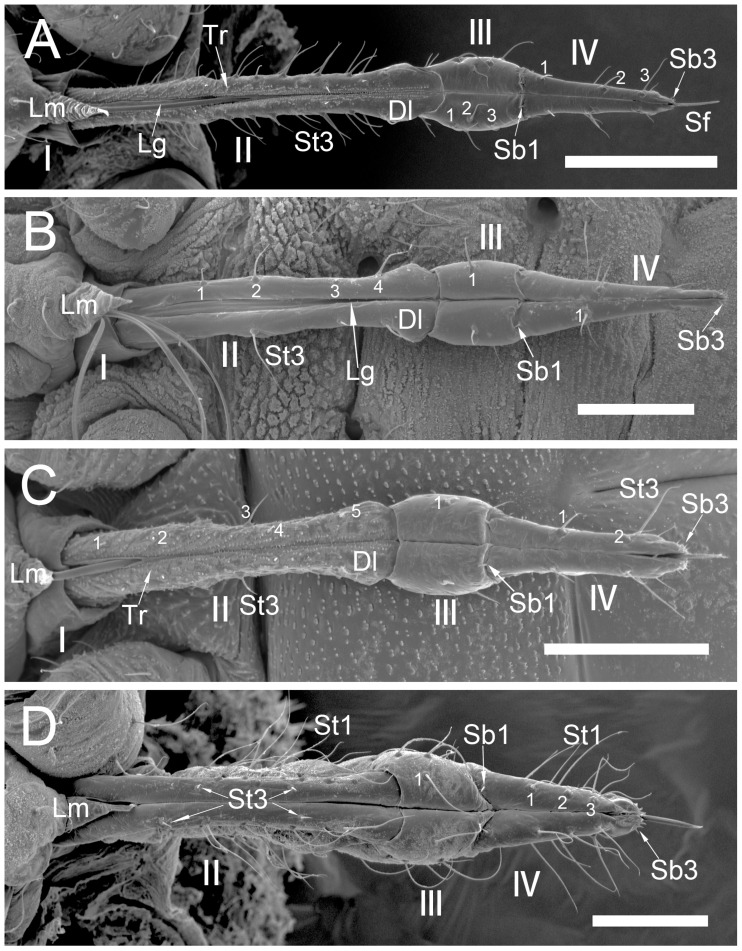
Morphology of the labium and arrangement of the mechanosensilla St3, St1, Sb1, and Sb3 from a ventral view. (**A**) *Uroleucon* sp. (**B**) *G. betulae*. (**C**) *M. cerasi*. (**D**) *P. juglandis*. Abbreviations: I–IV, different labium segments; Dl, dilation; Tr, tubercles; Lg, labial groove; Lm, labrum; Sf, stylet fascicle. Arabic numbers indicate the quantity of pairs and the arrangement of the different types of sensilla. Bars: (**A**) = 200 μm; (**B**–**D**) = 100 μm.

**Figure 10:**
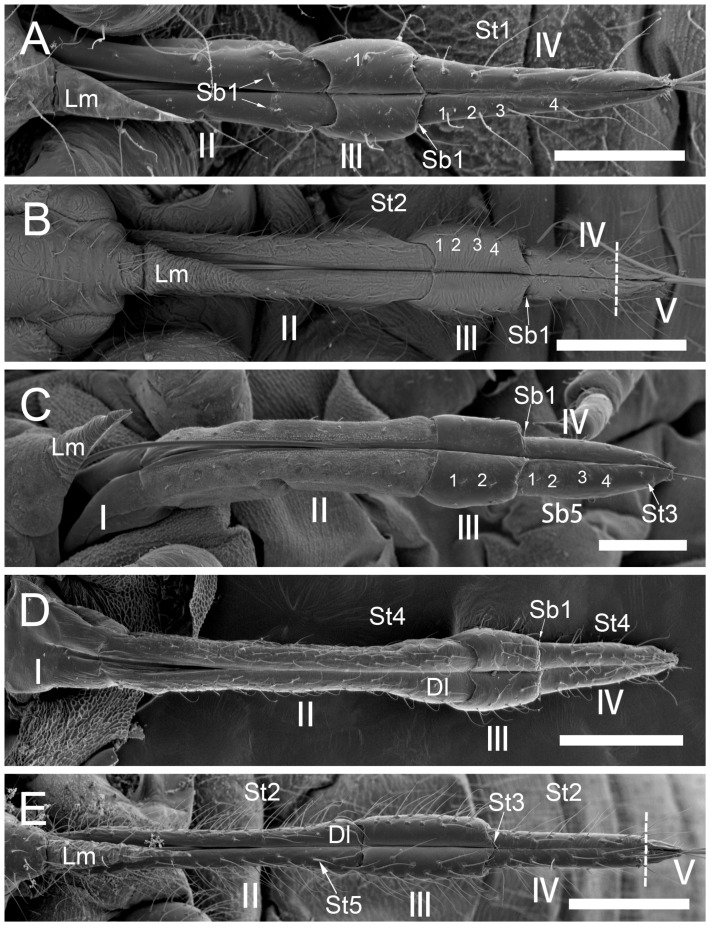
Morphology of the labium and arrangement of the mechanosensilla St1–St5, Sb1, and Sb5 from a ventral view. (**A**) *Chaitophorus* sp. (**B**) *L. roboris*. (**C**) *Forda* sp. (**D**) *P. cimiciformis*. (**E**) *Trama* sp. Abbreviations: I–V, different labium segments; Dl, dilation; Lm, Labrum. Arabic numbers: location and quantity of sensilla trichodea (sensilla basiconica in *Forda* sp.) in ventral side. White dotted line, boundary between the fourth and fifth segment. Bars: (**A**,**C**) = 100 μm; (**B**,**D**,**E**) = 200 μm.

**Figure 11:**
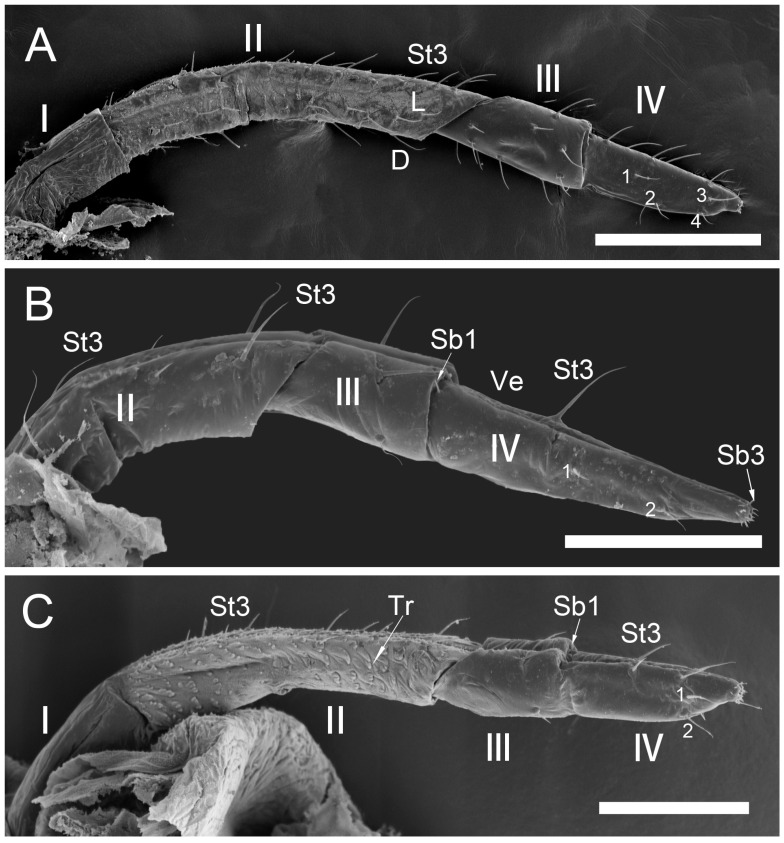
Morphology of the labium and arrangement of the mechanosensilla St3, Sb1 and Sb3 from lateral view. (**A**) *Uroleucon* sp. (**B**) *G. betulae*. (**C**) *M. cerasi*. Abbreviations: I–IV, different labium segments; Ve, ventral direction; L, lateral side; D, dorsal direction; Tr, tubercles; Arabic numbers: location and quantity of sensilla St3 in lateral side (fourth segment). Arabic numbers indicate the quantity the arrangement of the different types of sensilla. Bars: (**A**) = 200 μm; (**B**,**C**) = 100 μm.

**Figure 12:**
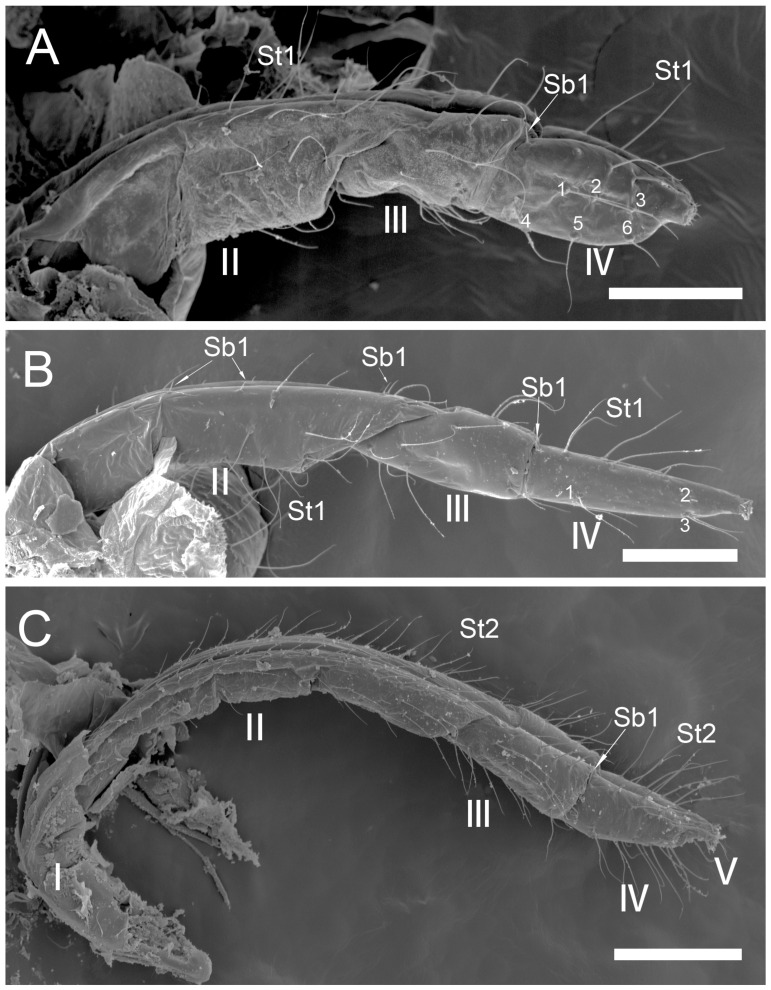
Morphology of the labium and arrangement of the mechanosensilla from lateral view. (**A**) *P. juglandis*. (**B**) *Chaitophorus* sp. (**C**) *L. roboris*. Abbreviations: I–V, different labium segments; Arabic numbers indicate location and quantity of pairs sensilla St1 in lateral side (fourth segment). Bars: (**A**,**B**) = 100 μm; (**C**) = 200 μm.

**Figure 13:**
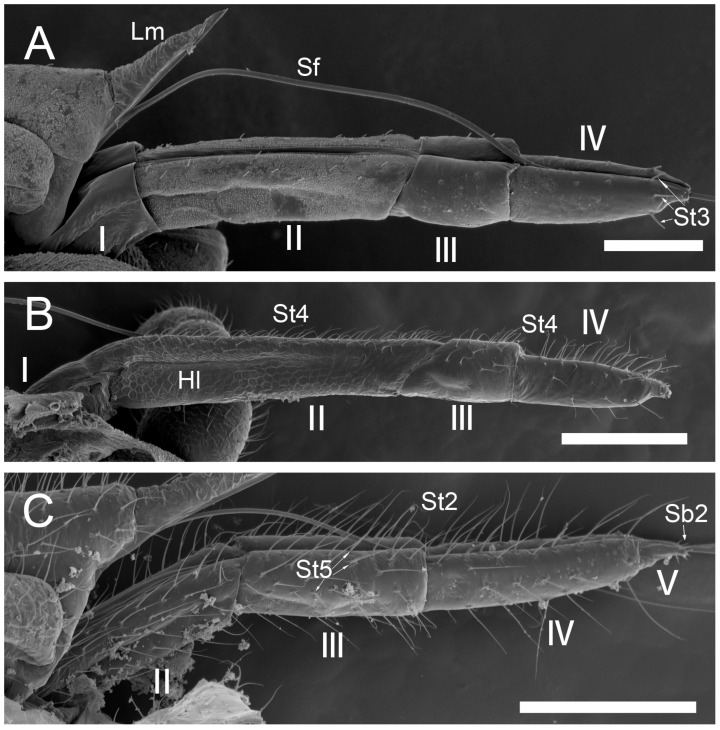
Morphology of the labium and arrangement of the mechanosensilla from lateral view. (**A**) *Forda* sp., sensilla St3. (**B**) *P. cimiciformis*, sensilla St4. (**C**) *Trama* sp., sensilla St2, St5, and Sb2. Abbreviations: I–V, different labium segments; Hl, honeycomb-like structure; Lm, labrum; Sf, tylet fascicle. Bars: (**A**) = 100 μm; (**B**,**C**) = 200 μm.

**Figure 14:**
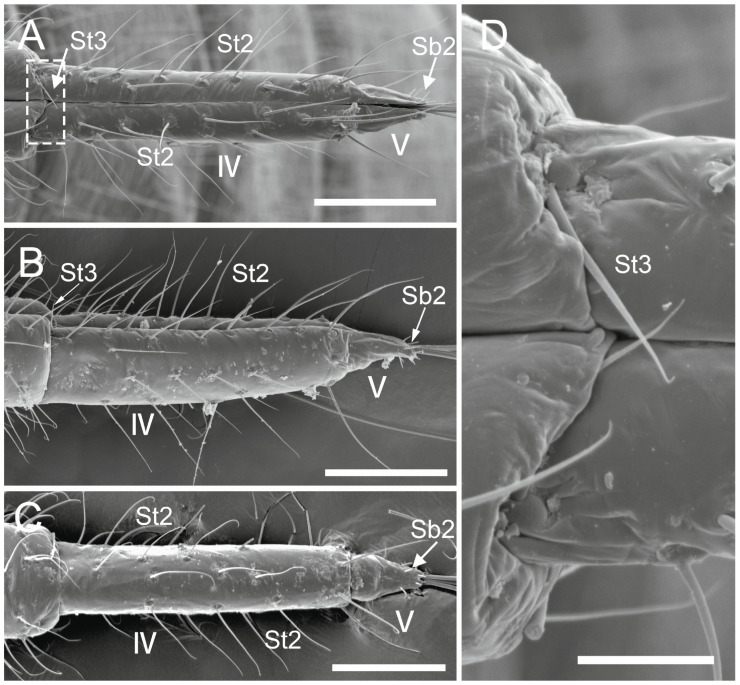
Sensilla of the fourth and fifth labium segment of *Trama* sp. (**A**) Ventral view, sensilla in base of fourth segment St3, IV segment with St2, V segment, Sb2 on the apex. (**B**) Lateral view, sensilla St3, St2, Sb2. (**C**) Dorsal view, IV segment with St2, V segment, Sb2 on the apex. (**D**) Enlarged view of the fourth segment base (white dotted box) in (**A**) showing a pair of sensilla trichodea 3 (St3). Bars, (**A**–**C**) = 100 μm; (**D**) = 20 μm.

**Figure 15:**
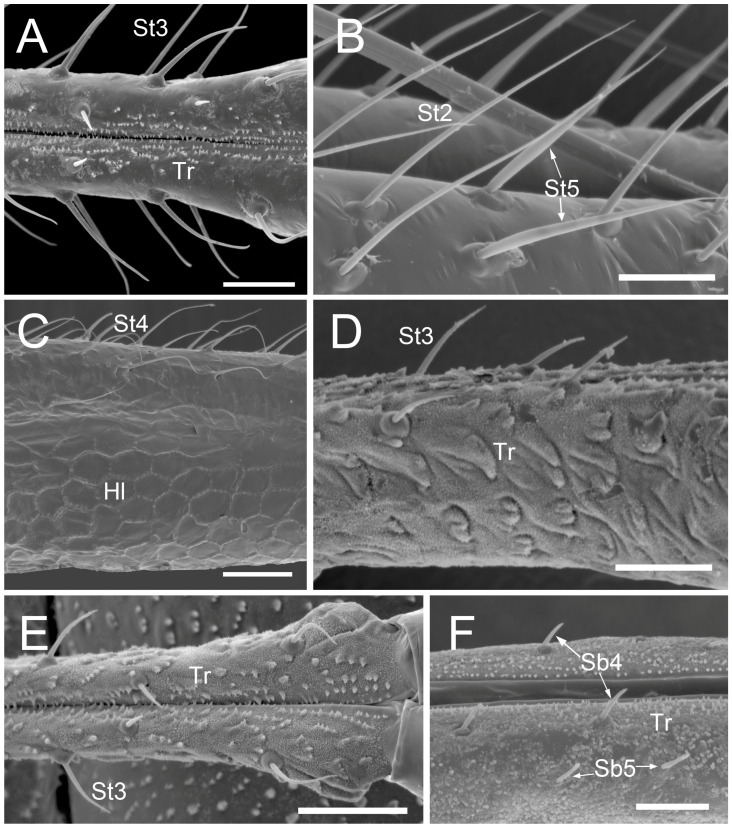
Second segment of labium showing some special structures and sensilla. (**A**) *Uroleucon* sp., ventral view, sensilla St3 and tubercles (Tr). (**B**) *Trama* sp., showing sensilla St2 and St5. (**C**) *P. cimiciformis* lateral view showing honeycomb-like (Hl) patterns and sensilla St4. (**D**,**E**) Lateral and ventral view of *M. cerasi* showing different shape tubercles (Tr) and sensilla St3. (**F**) *Forda* sp. ventral view showing distribution of sensilla Sb4 and Sb5 and tubercles (Tr). Bars, (**A**,**C**,**E**) = 30 μm; (**B**) = 10 μm; (**D**,**F**) = 20 μm.

**Figure 16:**
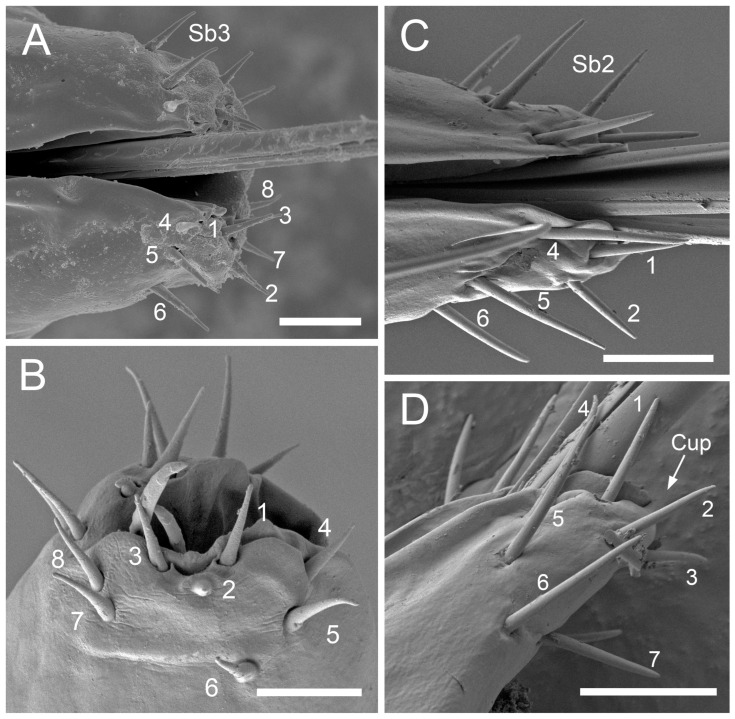
Types, numbers, and arrangement of the labial tip sensilla. (**A**) *M. cerasi*, sensilla Sb3 (**B**) *Uroleucon* sp., sensilla Sb3. (**C**,**D**) *Trama* sp., sensilla Sb2. Arabic numbers indicate the location and quantity of sensilla Sb2 and Sb3 from one side of the labial tip. Abbreviations: Cup, cuticular processes. Bars: (**A**,**B**) = 5 μm; (**C**,**D**) = 10 μm.

**Figure 17:**
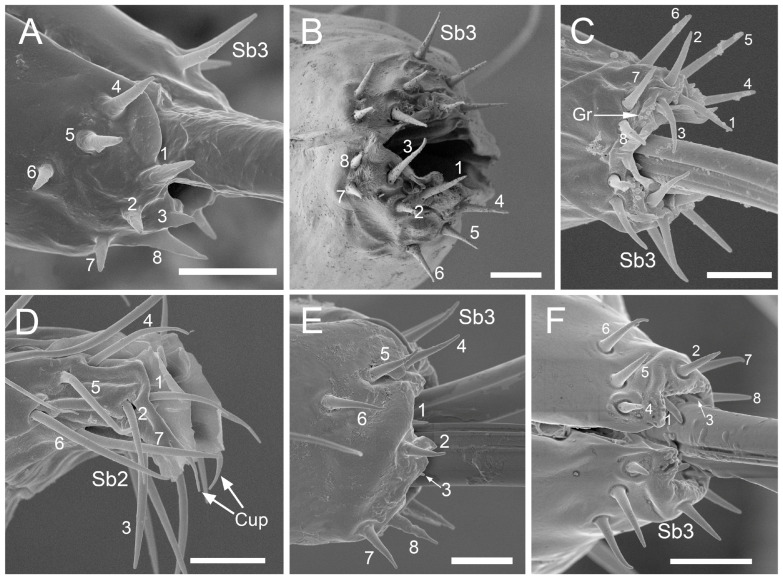
Types, numbers and arrangement of the labial tip sensilla Sb3 and Sb2. (**A**) *G. betulae*. (**B**) *P. juglandis*. (**C**) *Chaitophorus* sp. showing granular cuticular protrusions (Gr). (**D**) *L. roboris*., seven pairs of sensilla Sb2. (**E**) *Forda* sp. (**F**) *P. cimiciformis*. Arabic numbers indicate the location and quantity of sensilla Sb2 and Sb3 from one side of the la-bial tip. Abbreviations: Cup, cuticular processes. Bars: (**A**–**C**,**E**) = 5 μm; (**D**,**F**) = 10 μm.

The comparative analysis of the labrum, labium, and sensilla types, as well as the distribution of sensilla in select aphid species (Figure 1, Figure 2, Figure 3, Figure 4, Figure 5, Figure 6, Figure 7, Figure 8, Figure 9, Figure 10, Figure 11, Figure 12, Figure 13, Figure 14, Figure 15, Figure 16 and Figure 17), reveals both shared features and notable differences that likely reflect each species’ evolutionary adaptation to its ecological niche. For general orientation, Figure 2 of the entire aphid head and mouthparts is presented, with details and axes from specific mouthparts shown in the following Figure 4, Figure 5, Figure 6, Figure 7, Figure 8, Figure 9, Figure 10, Figure 11, Figure 12, Figure 13, Figure 14, Figure 15, Figure 16 and Figure 17. Terminology and classification of the sensilla are presented in Figure 3.

### 3.1. Morphological Type of the Sensilla

Insect sensilla occur in a wide variety of forms that differ conspicuously in their external morphology. The current classification is based on morphological characteristics, including shape, structural surface of the sensilla, type of socket, length (Table 1), and width of the sensilla stalk.

The labium of aphids bears various mechanosensilla typically cover its entire surface and are grouped apically into specific sensory fields. These sensilla are referred to as NP (non-porous) with flexible sockets (fs), which allow for deformation of the sensillum. The most abundant and widespread hair-like mechanosensilla are commonly referred to as sensilla trichodea or chaetica. Another group of mechanosensilla forms sensilla basiconica, which are shorter and stouter than hair-like sensilla.

#### 3.1.1. Sensilla Trichodea (St1–St5)

- Sensilla trichodea 1 (St1) and 2 (St2) are extremely slender, often with curved and sharply pointed tips, and are situated in sockets in hemispherical cuticular protrusions (Figure 3 and Figure 4A,B). These two types are morphologically similar; however, sensilla trichodea 2 can be distinguished by the presence of longitudinal grooves on their surface (Figure 3 and Figure 4B).
- Sensilla trichodea 3 (St3) and 4 (St4) resemble sensilla trichodea 1 and 2 but are comparatively shorter and thicker (Figure 3 and Figure 4C,D). These four types (St1–4) represent the most widely distributed sensilla among aphids. In species possessing them, they are generally present on the second, third, and fourth labial segments, except for sensilla St3 in *Forda* sp., which are restricted to the fourth segment.
- Sensilla trichodea 5 (St5) are also situated in sockets atop hemispherical cuticular protrusions and exhibit a smooth surface devoid of longitudinal grooves. They are distinctly swollen and thickened from the base to the mid-region (Figure 3 and Figure 4E). This sensillum type is presently observed in *Trama* sp.

#### 3.1.2. Sensilla Basiconica (Sb1–SB5)

- Sensilla basiconica 1 (Sb1) are straight, elongated-conical sensilla located in sockets on hemispherical cuticular protrusions (Figure 3 and Figure 5A). They are the most common type of sensilla basiconica, occurring at the base of the labrum in all examined species. Additionally, they occur in pairs at the base of the fourth labial segment, except in *Trama* sp.
- Sensilla basiconica 2 (Sb2) and 3 (SB3) are specialised sensilla located at the tip of the labium. They are conical in shape and situated within cuticular pits, exhibiting smooth surfaces lacking longitudinal grooves (Figure 3 and Figure 5B,C). Both types are arranged in pairs along the sides of the labial groove. Sensilla Sb2 are more slender and sometimes curved compared with sensilla Sb3 and are found only in two species of Lachninae (Figure 5C).
- Sensilla basiconica 4 (Sb4) are located in sockets on hemispherical cuticular protrusions. They are elongated-conical with longitudinal grooves and possess sharply pointed, slightly curved tips (Figure 3 and Figure 5D).
- Sensilla basiconica 5 (Sb5) are also located in sockets on hemispherical cuticular protrusions. Their general shape resembles that of sensilla basiconica 4; however, the tip is blunt, and the surface lacks longitudinal grooves (Figure 3 and Figure 5E). These two types are found exclusively on the labial surface of *Forda* sp.
- All sensilla bases recorded in this study possess flexible sockets (Figure 4F,G), indicating their capacity for a certain degree of movement.

### 3.2. Morphological Description of Labrum, Labium, and Sensilla in Particular Species of Aphids

***Uroleucon* sp.,** (Aphidinae)—species feeding on green shoots of herbaceous plants.
  - **Labrum** (Figure 6A and Figure 7A): Approximately 188.5 μm in length and 43.2 μm in width at the base (Table 2), presenting a triangular shape with transverse folds on the surface but no sensilla. A pair of sensilla basiconica 1 (Sb1) (Figure 3) is present at the base of the labrum, closely adjoined to the lateral sides. The stylet bundle extends from the base of the labrum posterior side, and a groove is present to accommodate and secure the stylet fascicle (Sf).
  - **Labium** (Figure 9A, Figure 11A, Figure 15A, and Figure 16B): Measures approximately 969.0 μm in length and is divided into four segments (I–IV), with the second segment being the longest and the third the shortest (Figure 9A and Table 3). The first segment is a soft, membranous, sleeve-like structure that partially envelops the second segment. Its surface exhibits folds and lacks sensilla. The second segment is approximately 441.4 μm long, with nearly uniform width at both ends in ventral view and a slight dilation distally. This segment has numerous sensilla (St3), at least a dozen pairs, primarily distributed along both sides of the labial groove and the lateral surfaces (Figure 9A and Figure 11A). In addition to sensilla, the surface of the second segment is covered with spiniform tubercles (Tr) (Figure 9A and Figure 15A), most densely arranged in band-like patterns along the labial groove. The third segment measures approximately 183.1 μm in length and appears trapezoidal in lateral view (Figure 11A). It has eight pairs of sensilla St3 (Figure 9A), with three pairs located ventrally (nos. 1–3) and the remaining on the lateral (L) and dorsal (D) surfaces (Figure 11A). The fourth segment is approximately 199.2 μm long and conical in shape, tapering distinctly toward the apex. This segment grows seven pairs of sensilla St3, distributed as follows: three pairs ventrally (Figure 9A, nos. 1–3), two pairs laterally, and two pairs dorsally (Figure 11A and Table 3). A pair of sensilla Sb1 is present at the ventral base of this segment (Figure 9A), while the tip grows eight pairs of sensilla Sb3 (Figure 9A and Figure 16B). These sensilla Sb3 are arranged in two groups: three pairs at the foremost tip (nos. 1–3) and five pairs peripherally (three pairs ventrally, nos. 4–6, and two pairs dorsally, nos. 7,8) (Figure 16B).

**Table 2:** Comparison of labrum features across species.

Species	Labrum Length (μm)	Labrum Width at Base (μm)	Shape	Surface Features	Sensilla Type and Location
*Uroleucon* sp.	188.5	43.2	Triangular	Transverse folds	Sb1 at base, lateral sides
*G. betulae*	128.0	44.5	Triangular	Surface folds	Sb1at base, lateral sides
*M. cerasi*	88.8	23.8	Triangular	Transverse folds	Sb1at base, lateral sides
*P. juglandis*	96.5	32.6	Triangular	Transverse folds	Sb1at base, lateral sides
*Trama* sp.	311.1	55.6	Conical	Groove, folds	Sb1at base, lateral sides, 3 pairs St2 on dorsal surface
*Chaitophorus* sp.	138.3	36.2	Triangular	Surface folds	Sb1 at base, lateral sides
*L. roboris*	358.1	66.6	Elongated Triangular	Transverse folds	Sb1at base, lateral sides
*Forda* sp.	185.2	52.2	Conical	Groove, folds	Sb1at base, lateral sides
*P. cimiciformis*	240.0	62.0	Elongated Triangular	Transverse folds	Sb1at base, lateral sides

**Table 3:** Comparison across the descriptions of the different aphid species, focusing on the features of their labium and the distribution of sensilla.

Species	Labium Length (μm)	Number of Segments	Segment 2 Length (μm)	Segment 2 Features	Segment 3 Length (μm)	Segment 3 Features	Segment 4 Length (μm)	Segment 4 Features	Sensilla (Segment-Wise)
*Uroleucon* sp.	969.0	4	441.4	Dilation spiniform tubercles, St3	183.1	Trapezoidal, St3	199.2	Conical, Sb1 at base, Sb3 at tip	Segment2: Numerous sensilla (St3), at least a dozen pairs, (both sides, labial groove). Segment 3: 8 pairs St3. Segment 4: 7 pairs St3, Sb1, Sb3.
*G. betulae*	585.1	4	265.1	Tubular, slight dilation sparse St3	86.7	Short, St3	176.3	Conical, five St3, Sb1	Segment 2: Sparse St3. Segment 3: 2 pairs St3. Segment 4: 5 St3, Sb1, Sb3.
*M. cerasi*	512.5	4	218.3	Dilation, numerous spiniform tubercles, St3	91.8	Short, stout, St3	125.9	Conical, St3, Sb1, Sb3	Segment 2: 5 pairs St3, spiniform tubercles. Segment 3: 2 pairs St3. Segment 4: 4 pairs St3, Sb1, Sb3.
*P. juglandis*	566.2	4	280.4	Short, stout, St1, St3	125.6	Short, St1	157.4	Conical, St1 Sb3	Segment 2: 17 pairs St1, 2 pairs St3 Segment 3: 10 pairs St1 Segment 4: 9 pairs St1, Sb1, Sb3.
*Trama* sp.	1287.0	5	572.1	Distal dilation dense, St2, St5	243.9	Narrow, elongated, numerous, St2, St5	249.9	Distal constriction, densely covered with St2, St3 = Sb1 St5	Segment 2: Densely covered St2. 1 pair St5; Segment 3: 30 pairs St2, 2–3 pairs St5. Segment 4: Densely covered St2, 1–2 pairs St5. Segment 5: 7 pairs Sb2
*Chaitophorus* sp.	687.3	4	335.6	Uniform, St1,Sb1	144.2	Trapezoidal, St1	196.7	Conical, St1, Sb1, Sb3	Segment 2: 10 pairs St1, 4 pairs of Sb1. Segment 3: 6 pairs St1. Segment 4: 6–7 pairs St1, Sb1, Sb3.
*L. roboris*	1446.0	5	883.9	Soft, slight thickening, dense St2	228.9	Abundant St2	200.6	Distal constriction, numerous St2	Segment 2: Numerous St2. Segment 3: 4 pairs St2. Segment 4: 14 pairs St2, Sb1, Sb3. Segment 5: Conical, 7 pairs Sb2
*Forda* sp.	734.2	4	329.1	Tubular, slight dilation Sb4, Sb5	122.5	Rectangular, Sb5	181.3	Conical, St3, Sb5, Sb1	Segment 2: 14 pairs Sb3, Sb4. Segment 3: 4 pairs Sb5. Segment 4: 4 pairs Sb5, 3 pairs St3; Sb3.
*P. cimiciformis*	1074.0	4	570.6	Distal dilation sparse, short spiniform tubercles, St4	169.3	Trapezoidal, St4	236.9	Conical, St4, Sb3	Segment 2: 36 pairs St4,. Segment 3: 13 pairs St4. Segment 4: 14 pairs St4, Sb1, Sb3

***Glyphina betulae*** (Linnaeus, 1758) (Thelaxinae)—species feeding on green tips of birch twigs.
  - Labrum (Figure 6B and Figure 7B): Approximately 128.0 μm in length and 44.5 μm in width at the base (Table 2), presenting a triangular shape with surface folds but no sensilla. A pair of sensilla basiconica 1 is present at the base, closely adjoined to the lateral sides. The stylet fascicle extends from the base of the labrum (Figure 7B).
  - Labium (Figure 9B, Figure 11B and Figure 17A): Measures approximately 585.1 μm in length and is divided into four segments (I–IV) with the second segment being the longest and the third the shortest (Table 3). The first segment is a soft, sleeve-like structure that can partially envelop the second segment, with no sensilla on its surface. The second segment is approximately 265.1 μm long, tubular in shape, and exhibits a distinct distal dilation (Dl) in ventral view. This segment has very few sensilla, with only four pairs of sensilla trichodea 3 distributed along both sides of the labial groove (Lg). The rest of the segment is largely smooth apart from minor folds. The third segment measures approximately 86.7 μm in length and appears trapezoidal in lateral view. It has two pairs of sensilla St3, with one pair located ventrally on both sides of the labial groove (Figure 9B) and the other pair dorsally (Figure 11B). The fourth segment is approximately 176.3 μm long and conical in shape, showing only slight constriction at the apex without distinct tapering. This segment also grows few sensilla St3, with only three pairs present: one pair ventrally (Ve) and two pairs laterally (L, nos. 1,2). A pair of sensilla basiconica 1 is located at the ventral base of this segment (Figure 11B), while the tip grows eight pairs of sensilla Sb3 (Figure 11B and Figure 17A). The sensilla Sb3 are arranged in two groups: three pairs at the foremost tip and five pairs peripherally (three pairs ventrally and two pairs dorsally) (Figure 17A).

***Myzus cerasi*** (Fabricius, 1775) (Aphidinae)—species feeding on leaves and young shoots of cherry and sweet cherry trees
  - Labrum (Figure 6C and Figure 7C): Approximately 88.8 μm in length and 23.8 μm in width at the base (Table 2), presenting a triangular shape with transverse folds on the surface but no sensilla. A pair of sensilla Sb1 is present at the base of the labrum, closely adjoined to the lateral sides. The stylet fascicle extends from the base of the labrum, and a posterior labrum groove is present to accommodate and secure it.
  - Labium (Figure 9C, Figure 11C, Figure 15E,F and Figure 16A): Measures approximately 512.5 μm in length and is divided into four segments I-IV), with the second segment being the longest and the third the shortest (Table 3). The first segment is a soft, sleeve-like structure that can partially envelops the second segment, with no sensilla on its surface. The second segment is approximately 218.3 μm long and exhibits a distinct distal dilation (DI) in ventral view. The surface of this segment is covered with numerous spiniform tubercles (Tr), which are arranged in band-like patterns (Figure 9C and Figure 15D,E). Those on both sides of labial groove are small and densely distributed, while those on lateral and dorsal surfaces are larger, more sparsely distributed, and exhibit more complex shapes. Sensilla on this segment are mainly concentrated ventrally, consisting of five pairs of sensilla St3: three pairs adjacent to the labial groove are shorter (nos. 1–3), and two pairs farther from the groove are longer (nos. 4,5) (Figure 9C). The third segment measures approximately 91.8 μm in length, appearing short and stout. It has only two pairs of sensilla St3, located ventrally and dorsally, with the ventral pair being longer. The fourth segment is approximately 125.9 μm long and conical in shape, without distinct constriction at the apex. It grows four pairs of sensilla St3, which can be clearly divided into two groups: one pair located ventrally at the mid-length of the segment (no 1) and three pairs near the tip (distributed ventrally no 2, laterally no 3, and dorsally no 4, one pair each) (Figure 9C and Figure 11C). A pair of sensilla Sb1 is present at the ventral base of this segment (Figure 9C), while the tip grows eight pairs of sensilla Sb3. These sensilla basiconica are arranged in two groups: three pairs at the foremost tip and five pairs peripherally (three pairs ventrallyn nos. 1,3,4 and five pairs dorsally, nos. 2, 5–8) (Figure 16A).

***Panaphis juglandis* (Goeze 1778)** (Calaphidinae)—species feeding on walnut leaves, on veins.
  - Labrum (Figure 6D and Figure 7D): Approximately 96.5 μm in length and 32.6 μm in width at the base (Table 2), presenting a triangular shape with transverse folds on the surface but no sensilla. A pair of sensilla basiconica 1 is present at the base of the labrum, closely adjoined to the lateral sides.
  - Labium (Figure 9D, Figure 12A, and Figure 17B): Measures approximately 566.2 μm in length and is divided into four segments (I–IV), with the second segment being the longest and the third the shortest (Table 3). The first segment has a smooth surface with no sensilla. The second segment is relatively short and stout, measuring approximately 280.4 μm in length, with no distinct dilation at the distal end. This segment grows numerous sensilla St1 (approximately 17 pairs), primarily distributed on the lateral surfaces. Additionally, two pairs of sensilla St3 are symmetrically arranged on the ventral surface (Figure 9D). The third segment is approximately 125.6 μm in length and has ten pairs of sensilla trichodea 1, with one pair located ventrally and the remaining pairs distributed on the lateral and dorsal surfaces. The fourth segment is approximately 157.4 μm long, triangular in ventral view, relatively short and stout, with a distinct constriction and tapering at the tip. It grows nine pairs of sensilla St1, with three pairs each on the ventral (nos. 1–3) (Figure 9D), lateral (nos. 1–3), and dorsal (nos. 4–6) surfaces (Figure 12A). A pair of sensilla Sb1 is present at the ventral base of this segment, while the tip has eight pairs of sensilla Sb3 (Figure 9D). The tip sensilla basiconica are arranged in two groups: three pairs at the foremost tip and five pairs peripherally (three pairs ventrally and two pairs dorsally) (Figure 17B).

***Chaitophorus* sp.,** (Chaitophorinae)—species feeding on leaves and young shoots of aspen.
  - Labrum (Figure 6E and Figure 7E): Approximately 138.3 μm in length and 36.2 μm in width at the base (Table 2), presenting a triangular shape with surface folds but no sensilla. A pair of sensilla basiconica 1 is present at the base of the labrum, closely adjoined to the lateral sides.
  - Labium (Figure 10A, Figure 12B, and Figure 17C): Approximately 687.3 μm in length and consists of five segments (I–IV), with the third segment being the shortest and the second segment the longest (Table 3). Sensilla are distributed on all segments except the first. The second segment measures approximately 335.6 μm in length, with nearly uniform width at both ends. Its surface grows two types of sensilla: sensilla St1 and sensilla Sb1. There are ten pairs of sensilla St1, with three pairs each along the labial groove and lateral surfaces (Figure 10A) and four pairs on the dorsal surface (Figure 10A and Figure 12B). The third segment measures approximately 144.2 μm in length and appears trapezoidal in lateral view (Figure 12B). This segment has only six pairs of sensilla St1: one pair along labial groove (no 1) (Figure 10A), two pairs laterally, and three pairs dorsally (Figure 12B). The fourth segment is approximately 196.7 μm long and conical in shape, without distinct constriction or tapering at the tip. It has seven to eight pairs of sensilla St1, distributed as follows: four pairs along both sides of the labial groove (nos. 1–4) (Figure 10A), one pair laterally (no 2), and three pairs dorsally (nos. 1–3) (Figure 12B). A pair of sensilla Sb1 is present at the ventral base of this segment, while the tip grows eight pairs of sensilla Sb3. These sensilla are arranged in two groups: three pairs at the foremost tip and five pairs peripherally (three pairs ventrally and two pairs dorsally) (Figure 17C). Granular cuticular protrusions are present around the three foremost pairs of sensilla basiconica.

***Lachnus roboris*** (Linnaeus, 1758) (Lachninae)—species feeding on woody oak twigs.
  - Labrum (Figure 6F and Figure 7F): Approximately 358.1 μm in length and 66.6 μm in width at the base (Table 2), presenting an elongated triangular shape with transverse folds on the surface but no sensilla. A pair of sensilla basiconica 1 (Figure 6F) is present at the base of the labrum, closely adjoined to the lateral sides. The stylet fascicle extends from the base of the labrum, and a ventral (posterior) groove is present to accommodate and secure it (Figure 7F).
  - Labium (Figure 10B, Figure 12C, and Figure 17D): Measures approximately 1446.0 μm in length and consists of five segments (I–V), with the third segment being shortest and second segment longest (Table 3). The first segment is a soft, elongated, membranous sleeve-like structure that can accommodate approximately half the length of the second segment. The first segment in the photo (Figure 10B) is not visible because it is pulled into the body of the aphids. When the second segment is partially retracted into the first, the labium bends into a fishhook-like shape (Figure 12C). The second segment measures approximately 883.9 μm in length, with a relatively soft surface, and is slightly thicker distally than basally. It has numerous sensilla trichodea 2 distributed on the ventral, lateral, and dorsal surfaces. The third segment measures approximately 228.9 μm in length and appears nearly trapezoidal in lateral view. Its surface grows abundant sensilla St2, with four pairs symmetrically arranged on both sides of labial groove (nos. 1–4) and the remainder located on the lateral and dorsal surfaces (Figure 12C). The fourth segment is approximately 200.6 μm in length, with a distinct distal constriction forming the fifth segment. It has a large number of sensilla St2 (approximately 14 pairs), distributed on all side surfaces (Figure 10B). A pair of sensilla Sb1 is present at the ventral base of the fourth segment, which is more slender compared to those found in most aphid species. The fifth segment is conical in shape, with seven pairs of sensilla Sb2 distributed at its tip, arranged in two groups: three shorter pairs at the foremost tip and four longer pairs peripherally. A pair of cuticular processes (Cup) is present at the apex of the labium, with moulting pores similar to sensilla Sb2 located at their bases (Figure 17D).

***Forda* sp.,** (Pemphiginae)—species and morph feeding on underground shoots and roots of grasses.
  - Labrum (Figure 6G and Figure 7G): Approximately 185.2 μm in length and 52.2 μm in width at the base (Table 2), convex conical in shape, with surface folds but no sensilla. A pair of sensilla Sb1 is present at the base of the labrum (Figure 6G), closely adjoined to the lateral sides. The ventral surface is flat, with a groove accommodating the stylet fascicle (Figure 7G).
  - Labium (Figure 10C, Figure 13A, Figure 15F, and Figure 17E): Measures approximately 734.2 μm in length and consists of four segments (I–IV), with the third segment being the shortest and the second segment the longest (Table 3). The first segment is a membranous, sleeve-like structure, allowing partial retraction of the second segment into it. Its surface is smooth, with no sensilla (Figure 10C). The second segment is approximately 329.1 μm in length, is tubular in shape, and is slightly thicker distally than basally. Only the ventral surface has sensilla, including 14 pairs of sensilla Sb4 and Sb5. In the examined specimens, a row of sensilla basiconica 4 is distributed adjacent to the labial groove, while sensilla Sb5 are located farther from the groove. Dense, short spiniform tubercles (Tr) are distributed on both sides of the labial groove, arranged in distinct band-like patterns (Figure 15F). The third segment measures approximately 122.5 μm in length and appears nearly rectangular in dorsal, lateral, and ventral views (Figure 10C and Figure 13A). Its surface is smooth and grows four pairs of sensilla Sb5, with two pairs on the ventral surface and two pairs on the lateral surface (nos. 1,2). The fourth segment has a smooth surface, measures approximately 181.3 μm in length, and is conical in shape with constriction at the apex. Four pairs of sensilla Sb5 are distributed on both sides of the ventral labial groove of this segment, and three pairs of sensilla trichodea 3 are located near the apex, with one pair each on the ventral, lateral, and dorsal surfaces (Figure 10C). A pair of sensilla Sb1 is present at the ventral base of the fourth segment, while the tip has eight pairs of sensilla Sb3. These apical sensilla are arranged in two groups: three pairs at the foremost tip and five pairs peripherally (three pairs ventrally and five pairs dorsally) (Figure 17E).

***Paracletus cimiciformis*** von Heyden, 1837 (Pemphiginae)—species and morph feeding on grass roots, where they live with ants *Tetramorium* sp., feeding also on hemolymph of ant larvae.
  - Labrum (Figure 6H and Figure 7H): Approximately 240.0 μm in length and 62.0 μm in width at the base (Table 2), presenting an elongated triangular shape with transverse folds on the surface but no sensilla. A pair of sensilla Sb1 is present at the base of labrum, closely adjoined to the lateral sides (Figure 6H). The stylet fascicle extends from the base of the labrum, and a ventral groove is present to accommodate and secure it (Figure 7H).
  - Labium (Figure 10D, Figure 13B, Figure 15C, and Figure 17F): Measures approximately 1074.0 μm in length and consists of four segments, with the third segment being the shortest and the second segment the longest (Table 3). The first segment is a smooth, membranous structure that is broad at the base and tapers toward the apex. The second segment is approximately 570.6 μm in length, with a slightly dilated distal end (DI). It has a large number of sensilla trichodea 4 (approximately 36 pairs), distributed exclusively on the ventral surface (Figure 13B). On the lateral and dorsal surfaces of this segment, dense honeycomb-like (Hl) patterns are visible (Figure 15C), with each polygonal unit formed by interconnected granular protrusions. The ventral surface lacks such patterns and exhibits only sparse, short spiniform tubercles (Tr). The third segment measures approximately 169.3 μm in length and appears trapezoidal in lateral view. It has 13 pairs of sensilla St4, distributed on the ventral and lateral surfaces. The fourth segment measures approximately 236.9 μm in length and is conical in shape, tapering distinctly toward the apex. This segment grows sensilla St4, with approximately 14 pairs on the ventral surface and a smaller number (approximately 7 pairs) distributed on the lateral and dorsal surfaces (Figure 10D and Figure 13B). A pair of sensilla basiconica 1 is present at the ventral base of the fourth segment, while the tip grows eight pairs of sensilla Sb3. These sensilla are arranged in two groups: three pairs at the foremost tip and five pairs peripherally (three pairs ventrally and five pairs dorsally). Among the three foremost pairs, two are situated very close to the inner side of the labial groove, often with only their anterior portions visible (Figure 17F).

***Trama* sp.,** (Lachninae)—species feeding on roots and underground shoots of herbaceous perennials of the Asteraceae.
  - Labrum (Figure 8A–E): Approximately 311.1 μm in length and 55.6 μm in width at the base (Table 2), exhibiting a generally conical shape. The ventral surface is relatively flat (Figure 8A), with a groove accommodating the protruding stylet fascicle from the base (Figure 8B,C). A pair of sensilla Sb1 is present at the base of the labrum, closely adjoined to the lateral sides, appearing more slender compared to other species (Figure 8D). In this species, the dorsal (anterior) surface of the labrum, in addition to folds, grows three pairs of sensilla St2 (Figure 8E), mainly concentrated in the middle part. This represents the only recorded aphid species with sensilla present on labrum.
  - Labium (Figure 10E, Figure 13C, Figure 14A–D, Figure 15B, and Figure 16C,D): Measures approximately 1287.0 μm in length, consists of five segments (I–V), and exhibits a slender, tubular form (Table 3). The labial surface, except for the first segment, grows a large number of sensilla. The first segment is a soft, sleeve-like structure that partially envelops the second segment. The second segment, the longest of the labium at approximately 572.1 μm, shows a slight distal dilation (DI) in ventral view. Its surface is densely covered with sensilla trichodea 2, primarily concentrated along both sides of the labial groove and the lateral surfaces. Additionally, one pair of sensilla St5 is located on the ventral surface near the distal end (Figure 10E and Figure 15B). The third segment measures approximately 243.9 μm in length, appears rectangular in lateral view, and is notably narrower and more elongated (length-to-width ratio > 2) compared to most aphids. Its surface grows approximately 30 pairs of sensilla St2 and 2–3 pairs of sensilla St5, mainly concentrated on the ventral and lateral surfaces (Figure 13C). The fourth segment is approximately 249.9 μm in length, with a distinct distal constriction that forms a secondary fifth segment at the tip. The fourth segment is also densely covered with sensilla St2, distributed on the ventral, lateral, and dorsal surfaces (Figure 13C and Figure 14A–C). Additionally, 1–2 pairs of sensilla St 5 are present on the ventral surface (Figure 15B). A pair of sensilla St3 is located at the ventral base of this segment, which distinctly differs from the sensilla basiconica typically found in this position in most aphids (Figure 14D). The fifth segment is conical in shape, with seven pairs of sensilla Sb2 distributed at its tip, arranged in two groups: three shorter pairs at the foremost tip and four longer pairs peripherally (Figure 16C,D). A pair of cuticular processes (Cup) is present at the apex of the labium, with moulting pores similar to sensilla Sb2 located at their bases. The length statistics of the sensilla are summarised in Table 1.

#### 3.2.1. Labrum Comparison

The labrum is triangular and plane-like in most species, except for *Trama* sp. and *Forda* sp., which have a labrum more convex on the dorsal side, “conical-shaped”. Length ranges from 88.8 μm (*M. cerasi*) to 358.1 μm (*L. roboris*) and width varies from 23.8 μm (*M. cerasi*) to 66.6 μm (*L. roboris*) (Table 2). Labrum length increases markedly in taxa feeding on woody or subterranean tissues (e.g., *L. roboris*, *Trama* sp., *P. cimiciformis*), suggesting scaling of rostral components with penetration depth and mechanical resistance of the host tissue.

Sensilla (Table 2): *Trama* sp. is the only species that exhibits sensilla on the labrum, specifically three pairs of sensilla trichodea (St2) on the dorsal surface, which is unique among the species listed. The other species (*Uroleucon* sp., *G. betulae*, *M. cerasi*, *P. juglandis*, *Chaitophorus* sp., *L. roboris*, *P. cimiciformis*) show no sensilla on the labrum surface, although all have a pair of sensilla basiconica (Sb1) at the base of the labrum, closely adjoined to the lateral sides.

#### 3.2.2. Labium Comparison

*Segment Number and Length*: Some studied species have a labium divided into four segments, although *Trama* sp. and *L. roboris* have five segments (both species belong to the Lachninae). The second segment is generally the longest across species, though its length varies significantly. For example: *Uroleucon* sp. 441.0 μm, *L. roboris*: 883.9 μm, *Trama* sp.: 572.1 μm (Table 3). The first segment is a membranous, sleeve-like structure in all species, with no sensilla, serving to partially envelop the second segment. Leaf- and shoot-feeding species (*Uroleucon* sp., *M. cerasi*, *P. juglandis*, *Chaitophorus* sp.) possess relatively short labium (≈500–900 µm). These labia show moderate sensilla densities, reflecting reduced mechanical demands when accessing superficial phloem. In turn, twig- and bark-feeding species (*L. roboris*) exhibit extremely elongated labia (>1400 µm), including a highly extensible first segment and densely sensillated second and third segments. This morphology facilitates penetration through lignified tissues and prolonged feeding. However, root feeder species (*Forda* sp., *P. cimiciformis*, *Trama* sp.) display intermediate to long labia (700–1300 µm) with pronounced sensilla specialisation (Sb4, Sb5, St5).

**Sensilla distribution:**
  - Across the examined aphid taxa, five types of sensilla trichodea (St1–St5) and five types of sensilla basiconica (Sb1–Sb5) are distinguished, varying in length (Table 1). Sensilla trichodea represent the most abundant (Table 3) and widely distributed sensillum type, whereas sensilla basiconica show stronger positional and taxon-specific constraints.
  - Sensilla (St1–St4) typically occur on the second to fourth labial segments. Sensilla St5 is restricted to the second segment in *Trama* sp., suggesting that this taxon has specific characters.
  - Sensilla (Sb1–Sb5) show greater functional differentiation. Sensilla Sb1 is conserved across all examined taxa except for *Trama* sp. and consistently occurs at the base of the labrum and the ventral base of the terminal labial segment, supporting its interpretation as a conserved proprioceptive/mechanosensory element. In contrast, Sb2–Sb3 exhibit restricted distributions to the apex of labium, but Sb4-Sb5 are associated in *Forda* sp. with all surfaces of the labium.
  - **Special features**: *Trama* sp. features cuticular processes (Cup) at the apex of the labium, which are also seen in *L. roboris*. Moreover, the last has distinct granular cuticular protrusions on the lateral and dorsal surfaces of the second segment, forming honeycomb-like patterns that are absent in other species.
  - **Conical and tapering segments:** The fourth segment tends to taper toward the apex and is often conical in shape for most species. This is especially true for *M. cerasi, P. juglandis, Trama* sp., *Forda* sp., *P. cimiciformis*, and *L. roboris*, while *Uroleucon* sp. and *G. betulae* show more smooth or less distinct tapering.

**Sensilla types and distribution patterns (Table 3)**
  - **Sensilla basiconica:** A pair of sensilla (Sb1) is located at the ventral base of the fourth segment of the labium in most species. Eight pairs of sensilla (Sb3) are found at the tip of the labium in the studied species except *Trama* sp. and *L. roboris*, which possess seven pairs of sensilla (Sb2). In *Forda* sp. the labial surface is covered by the sensilla basiconica Sb4 and Sb5, and only a few sensilla St3 are observed on the last segment.
  - **Sensilla trichodea**: Sensilla (St1), (St2), and (St3) are common in certain species, but St1 is generally found in higher numbers than St3, particularly on the ventral and lateral surfaces of *P. juglandis* and *Chaitophorus* sp. The common type of St2 sensillum was found in *L. roboris* and *Trama* sp., whereas *Uroleucon* sp., *G. betulae* and *M. cerasi* mainly exhibit St3 sensilla from the second to fourth segments. Trichodea sensilla St4 are notably found in *P. cimiiformis*, whereas St5 are restricted to *Trama* sp.
  - **Special variations**: In *Uroleucon* sp., *M. cerasi*, and *G. betulae*, sensilla St3 are highly concentrated in specific regions such as the ventral labial groove and lateral surfaces, with no other types of sensilla trichodea in the segments. *L. roboris* and *Trama* sp. stand out for the sheer number of sensilla trichodea 2 found on the segments, distributed symmetrically on all surfaces. Sensilla trichoidea St3 have been observed at the ventral base of the fourth segment in the *Trama* sp.

**Overall summary of comparison:**
  - **Size Variations**: Five-segmented labia occur in *L. roboris* and *Trama* sp., while the remaining species possess four-segmented labium.
  - **Sensilla Patterns**: *Trama* sp. is unique for its combination of sensilla (St2, St3, and St5) and sensilla (Sb2), along with the highest concentration of sensilla (St2) on its second and third segments.
  - **Unique Features**: Only *Trama* sp. and *L. roboris* contains a combination of cuticular processes and distinct granular protrusions, which sets it apart from the other aphids that mostly share basic sensory structures on the labium, particularly on the second segment.

## 4. Discussion

In the present study, we review and synthesise available knowledge on labrum and labium shape and labial mechanosensilla of aphids: their morphological characteristics, spatial distribution, hypothesised functions in feeding behaviour, and any variation among species. This will provide a foundation for subsequent empirical investigations of rostral mechanosensory systems in aphids.

The labrum across Sternorrhyncha exhibits a gradient of structural complexity that correlates strongly with feeding behaviour. Aphids have a simplified, small, usually triangular, and membranous labrum that is optimised for phloem-feeding efficiency and flexibility. However, some taxa possess a conical labrum and certain species, such as *Trama* sp., exhibit mechanosensilla. This is similar to the phenomena found in scale insects and whiteflies, which exhibit further reduction tied to their sedentary or highly specialised feeding modes [26]. In psyllids, the labrum is retained for mechanical penetration.

### 4.1. Labium Segmentation and Functional Morphology

Comparative analysis across the examined aphid taxa reveals clear evolutionary trends in labial segmentation that correspond closely to both phylogenetic position and ecological specialisation. The labium serves as the guiding and protective structure for the stylet bundle, and its segmentation pattern determines both the range of motion and the mechanical precision of stylet operation. Among the studied species, *Uroleucon* sp., *G. betulae*., *M. cerasi*, *P. juglandis*, *Forda* sp., and *P. cimiciformis* exhibit a four-segmented labium, representing the plesiomorphic and most widespread condition within the Aphidinae and related lineages of Aphididae [5,17,27]. Each segment differs in length, with the second consistently the longest—an arrangement that confers a balance between stability and manoeuvrability during feeding on soft plant tissues.

In contrast, *Trama* sp. and *L. roboris* possess a five-segmented labium, a derived configuration largely restricted to members of the Lachninae. In these forms, the apical (fourth) segment is secondarily subdivided [1,5]. This additional articulation likely restores, in part, an ancestral pattern of segmentation. Emeljanov [2] proposed that the primitive sternorrhynchan rostrum was three-segmented and that the four- and five-segmented conditions observed in extant aphids arose through secondary splitting of the distal segment. The present evidence supports this interpretation, implying that the five-segmented configuration in *Trama* and *Lachnus* represents a re-expression of an ancestral developmental pathway, now co-opted for mechanical refinement.

The occurrence of an additional segment in *Trama* sp. and *L. roboris*—both species feeding on mechanically resistant substrates such as roots and woody tissues—suggests that increased segmentation enhances flexibility and the precision of stylet control during deep penetration. Lachninae represent an ancestral lineage that includes numerous woody-tissue phloem feeders. The genus *Cinara* dominates species diversity, feeding exclusively on woody tissues and comprising the largest extant radiation of aphids on conifer hosts. Lachnini are less species-rich but utilise a broader range of woody angiosperm hosts, whereas Tramini form a distinct lineage that feeds on the roots of herbaceous angiosperms [28]. Comparable elongations have evolved independently in bark- or root-feeding aphids *Prociphilus* and *Phloeomyzus* species [29,30,31], supporting an interpretation of convergent functional elongation under mechanical constraints imposed by woody tissues. In *Trama* sp. and *L. roboris*, the basal labial segment in these taxa is notably membranous and flexible, forming a sheath that partially encloses the second segment. This configuration allows telescopic retraction and bending of the rostrum in multiple planes, depending on feeding posture and the orientation of the host surface.

A comparable mechanism has been described in *Stomaphis* species, in which the proximal segments retract within one another, allowing the labium to extend several times the body length without structural collapse [1]. The present observations of *L. roboris* show an analogous condition, where telescopic flexibility provides both reach and stability during stylet insertion through thick bark. Such design features suggest that segmentation complexity in these taxa primarily functions as a biomechanical adaptation for deep penetration and precise alignment of the stylet bundle, rather than as a taxonomic or phylogenetic peculiarity.

The typical four-segmented labium found *Eriosoma lanigerum* (Hausmann) (Pemphiginae) [32] and in most Aphidinae and Calaphidinae [3] appears optimal for feeding on soft, herbaceous tissues, where repeated short insertions require rapid repositioning and fine motor control. A shorter labium reduces leverage force and facilitates shallow penetration, minimising mechanical stress on the slender stylets [3,16]. Conversely, an elongated, five-segmented labium, characteristic of *Lachnus*, *Stomaphis*, and *Trama*, is found in a taxon that feeds on lignified or subcortical tissue. In this case, the rostrum must penetrate a substantial mechanical barrier and remain stable during prolonged ingestion.

Occasional reductions to three apparent segments, as reported for *Aphis citricola* van der Goot (Aphidinae: Aphidini) [33], probably result from the fusion of some segments. A phenomenon of three-segmented labium was also indicated in another aphid, e.g., *Euthoracaphis umbellulariae* (Essig) (Hormaphidinae: Nipponaphidini), which lives and feeds on the leaf undersides of plants of the Lauraceae [34]. Such fusions or divisions within the same family underscore the developmental plasticity of the aphid labium, which is capable of evolving through both segmental fusion and resegmentation in response to ecological demands.

Labium architecture has evolved as a dynamic system integrating flexibility, rigidity, and sensory precision—traits that together facilitate the extraordinary feeding specialisation and adaptive radiation characteristic of the Aphididae.

### 4.2. Sensillar Diversity and Distribution Patterns

The sensilla set across species demonstrates substantial diversity—both quantitative and topographical. Across all taxa examined, sensilla trichodea represent the dominant mechanosensory type, occurring on nearly all labial segments except the basal one. Their symmetrical arrangement along the ventral, lateral, and dorsal surfaces suggests a broad tactile function during probing. *Uroleucon* and *L. roboris* possess particularly dense arrays of sensilla trichodea 2 on the second segment, which are necessary for tactile function during feeding on different plant surfaces. Also, dense distributions were described by Wensler [15] and Tjallingii [16] at the ultimate rostral segment in *Aphis fabae* Scop. and *M. persicae*, where sensilla respond to stylet movement and cuticular strain.

*Trama* sp. is morphologically exceptional, exhibiting multiple trichoid sensilla types (St2, 3, 5) together with basiconic sensilla Sb1 and SB2, and even sensilla on the labrum itself—a rare feature in aphids. So far, a labrum covered with hairs has also been recorded in the genus *Stomaphis* (also Lachninae) [35], but these were not interpreted by the authors as trichoid sensilla, and their character and function in *Stomaphis* aphids remain unstudied. Trichoid and basiconic sensilla on the labium were also recorded in *Sinolachnus* (Lachininae), but not in such diversity [36]. The presence of sensilla (St2) on the ventral surfaces of the labium in *Trama* sp.and *L. roboris* may be used as the labium bends or retracts. *Trama* sp., which feeds on subterranean roots of herbaceous angiosperms, displays an even higher degree of sensory specialisation, including unique sensilla types (St5).

The apical sensory field, formed by concentric rings of eight pairs of sensilla Sb3 of varying length from 3.5 to −6.8 μm, was observed across seven studied species. Another pattern was observed, with seven pairs of Sb2. Their lengths range from 11.1 μm in *Trama* sp. to 16.5 μm in *L. roboris*. Similarly to *Cinara pilicornis* (Hartig), the apical end of the last rostral segment has seven pairs of (5.50–6.50 μm) in length and smooth sensilla basiconica (type III) [37]. Although *Trama* sp., *L. roboris*, and *C. pilicornis* represent a common subfamily (Lachninae), the last species was found to have other types of sensilla, but in the same number. The pattern of seven pairs of sensilla Sb3 has also been documented in *Pseudessigella brachychaeta* Hille Ris Lambers (Lachninae) [38], and this feature has also been seen in *Eriosoma lanigerum* (Eriosomatinae) (Hausmann) [32]. The pattern with the seven Sb3 is shared by species from different subfamilies, including Lachninae and Eriosomatinae, indicating that this trait is not strictly subfamily specific. Although variation exists in sensillum length and type among species such as *Trama* sp., L. *roboris*, and *C. pilicornis*, the conserved number and arrangement suggest a common functional role. These apical sensilla correspond to the labial sensory field described in other Hemiptera, supporting the view that this sensory structure is evolutionarily conserved and likely essential for assessing the host plant during feeding [15,16]. Meanwhile, the paired sensilla Sb1 located ventrally at the base of the apical segment are likely mechanoreceptive, monitoring stylet motion within the labial groove. Such conservative placement of the mentioned sensilla across diverse aphids underscores their fundamental role in mechanosensation. Meanwhile, the paired sensilla Sb1 located ventrally at the base of the apical segment are likely mechanoreceptive, monitoring stylet motion within the labial groove. Such conservative placement of the mentioned sensilla across diverse aphids underscores their fundamental role in mechanosensation.

Distinct surface textures—including spiniform tubercles, granular protrusions, and honeycomb-like sculpturing—were observed primarily on the second and third segments. In *M. cerasi* and *G. betulae*, the dense, banded spiniform tubercles along the labial groove may reinforce cuticle flexibility. In *L. roboris*, the honeycomb-like texture on the dorsal and lateral surfaces of the second segment likely enhances structural strength while preserving elasticity, a necessary balance for bark penetration. Similar microstructural modifications have been reported for other bark-feeding aphids [1]. Feeding on grass roots, *P. cimiciformis* also exhibits a polygonal, granular surface pattern, possibly linked to enhanced tensile resilience, particularly during subterranean interactions or ant-associated life stages. The additional cuticular processes (Cup) at the labial apex in *Trama* and *Lachnus* may influence the displacement of the stylets during root penetration.

### 4.3. Mechanosensory Function

The tip of the labium remains in contact with the substrate surface while the aphid feeds [39,40,41,42]. However, behavioural studies have shown that the palpating movements of the labium are generally performed during small-scale exploration of the plant surface [16]. The integration of various types of mechanosensory input on the mouthparts is probably essential for the successful recognition of plant surfaces in aphids [15,16]. Mechanosensilla provide feedback on the physical interaction between the stylet bundle and host tissues.

In the species examined in the present study, the basiconic Sb3 and Sb2, located mainly on the labial apex, probably do not detect plant chemical cues. Although these sensilla are similar in structure to other contact-chemoreceptive sensilla with dual function in many hemipteran insects [11], detailed observations did not confirm the presence of terminal pores. Therefore, their morphological features do not support a chemosensory function. The gustatory sensilla are probably associated with the pharynx, which comes into contact with phloem sap after the stylets penetrate the plant tissue in aphids [15]. This situation is similar to that observed in leafhoppers [43].

Previous studies have described similar peg-type receptors on the labium of the cabbage aphid *Brevicoryne brassicae* L. [41] and the peach aphid *M. persicae* (Sulzer) [44]. These peg receptors at the tip of the labium in aphids have been interpreted as contact chemoreceptors [41,45]. However, Forbes [46] suggested that they have the structure of tactile receptors because the pegs are innervated by a single dendrite. This sensory neuron likely does not have direct access to chemical stimuli through openings in the cuticle, unlike comparable pegs at the tip of the labium in *Dysdercus* and the milkweed bug *Oncopeltus fasciatus* (Dallas). Nevertheless, a chemoreceptive function of these pegs has been demonstrated electrophysiologically in the latter two species [47].

Ultrastructural studies conducted by Wensler [15] on the distal tip of the labium of *B. brassicae* revealed that the peg (basiconic) sensilla are mechanoreceptors. Each peg is innervated by a single sensory neuron that is anchored eccentrically to a basal cuticular tube and terminates in an electron-dense material dendritic sheath with densely grouped microtubules that form a tubular body at the base of the peg. According to Wensler [15], the peg sensilla at the labial apex detect surface contact (pressure) and surface profile. The bilateral symmetry of these peg receptor arrangements facilitates the detection of vein contours, which are preferred sites for settling and feeding.

Similar findings were reported by Tjallingii [16], who confirmed that in *B. brassicae*, both the tip sensilla and the sensilla (larger labial hairs) located in more proximal regions are innervated by a single dendrite with a tubular body, which indicates a mechanosensory role. The sensilla along the stylet groove are also likely to be mechanoreceptors. Based on the data of Wensler [15], Forbes [48], and Tjallingii [16], it can be concluded that the labium lacks a chemosensory function (gustatory or contact chemoreception).

By contrast, in other Sternorrhyncha, such as whiteflies (*Bemisia tabaci* (Gennadius) and *Trialeurodes vaporariorum* (Westwood)), there are two morphological subtypes of labial tip sensilla: the apical pore (UPP) and the subapical pore (UPS). These sensilla have been shown to function as chemoreceptors (gustatory) and bimodal mechano-chemoreceptors [26]. Likewise, in the Asian citrus psyllid (*Cacopsylla chinensis* (Yang &Li)), mechanosensilla are mainly distributed along the sides of the labial groove, while four pairs of bilaterally symmetrical apical labial sensilla basiconica (S. b. II) resemble the whitefly gustatory sensilla [19].

The present study showed a high density of sensilla (St2) on the second and third segments in *Trama* sp. and *L. roboris*. These sensilla probably facilitate fine-scale control of labial curvature and penetration depth, similar to the other type of sensilla (St1–St5) in other species. Moreover, proprioceptive sensilla (St3-in *Trama*, and in other species Sb1) are also present on the borders of the segments. These structures are likely homologous to the proprioceptive basiconic sensilla (Sb) or trichoid (St) observed in other hemipterans, which detect segmental deformation or curvatures [15,16].

Given the extreme elongation of the labium in trunk- and root-feeding taxa, precise sensory response is likely indispensable to prevent mechanical failure of the stylets or misalignment during feeding. The absence of trichoid sensilla on the basal segment, combined with its membranous flexibility, suggests that mechanosensory input is concentrated distally, near the stylet tip, where the risk of physical damage and the need for navigation are greatest.

The rostrum of aphids is complemented by a second sensory system associated with the mandibular stylets. Classic morphological studies have demonstrated that only the mandibular stylets are innervated, whereas the maxillary stylets lack neural elements. Forbes [17,49] reported nerve elements in the mandibular stylets of *Myzus persicae* and later provided a detailed account of stylet morphology and penetration mechanisms. These studies showed that only mandibular stylets contain paired dendrites with microtubule bundles. Similar observations were subsequently reported in *Rhopalosiphum maidis* (Fitch) (two axons and sensory dendrites within the central canal of each mandibular stylet), supporting the generality of innervation [50].

The innervation of mandibular stylets is consistent with a mechanosensory function. The paired dendrites likely detect mechanical resistance, tissue density, and stylet position during penetration and pathway selection through plant tissues. Mandibular receptors, as described in ultrastructural studies, are interpreted as mechanoreceptors involved in monitoring stylet movement and deformation at the labial tip. The presence of rigid receptor components, such as scolopales, further supports a role in detecting strain rather than chemical cues [17,47]. Although two dendrites per mandibular stylet appear to be the most common condition, variation exists. Forbes and Mullick [8] reported three dendrites in the mandibular canal of *Adelges piceae* (Ratzeburg) (Adelgidae), suggesting evolutionary plasticity in sensory capacity that may reflect differences in feeding substrate or penetration depth.

Together, these studies support a dual sensory system in aphids, integrating external mechanosensory input from distal labial sensilla with internal feedback from innervated mandibular stylets. This system is crucial for various types of penetrating feeding in aphids, where precise control of labial and mandibular movement is essential for successful feeding and mechanical integrity during movement of the maxillae.

### 4.4. Ecological and Evolutionary Implications

The combined morphological and sensilla evidence reveals a close correspondence between rostral architecture and feeding ecology among aphid taxa. Variation in labial length, segmentation, and sensilla composition reflects adaptive responses to the mechanical and spatial properties of host-plant tissues (Table 4). Herbaceous-feeding aphids exhibit short, four-segmented labium with moderate sensilla density—structures well suited for rapid exploratory probing of soft epidermal and mesophyll tissues. Such compact rostrum maximise manoeuvrability and sensory precision during frequent repositioning across plant surfaces. Conversely, if the attachment is too strong for plants, escape will be difficult. *Uroleucon* sp., for example, reacts instantly by falling away from the plant; extending its stylets takes as little as a second. Another taxon with short mechanosensilla (St3 and St4) but long labium, such as *Paracletus*, does not require this type of special protection because it smells like an ant and is treated like an ant [51].

In contrast, woody-feeding species such as *L. roboris* and *Stomaphis* spp. possess markedly elongated, five-segmented labium that facilitate stylet penetration through thick, lignified bark. These labia bear densely and evenly distributed trichoid sensilla, particularly on the middle segments, likely serving as mechanosensory guides to regulate labial curvature and penetration depth. The root-feeding forms (*Trama* sp., *Forda* sp.) display even greater sensilla diversity—including sensilla St5 and Sb4 and Sb5—together with localised cuticular reinforcement. These traits appear to represent biomechanical and sensory adaptations to the irregular, resistant, and heterogeneous substrates encountered below ground.

Such functional differentiation indicates that similar ecological pressures—especially substrate hardness, tissue depth, and feeding orientation—have repeatedly driven convergent modifications of the rostrum in distantly related aphid lineages. This pattern parallels trends in other Sternorrhyncha, such as *A. piceae* [8] and *D. vitifoliae* [9], where elongate stylets and specialised labial structures have evolved independently for bark or root feeding.

At a broader evolutionary scale, these morphological and sensory innovations exemplify adaptive radiation within the Aphididae, reflecting the aphid labium capacity to evolve as an integrated organ system. The repeated evolution of elongated, retractable labium in phylogenetically related taxa of the subfamily Lachninae (*Stomaphis, Lachnus* and *Trama*) and phylogenetically distant *Prociphilus* (Eriosomatinae) and *Phloeomyzus* (Phloeomyzinae) suggests that selective pressures imposed by host-plant anatomy—rather than shared ancestry—have been the primary drivers of these traits. Furthermore, the diversity of sensilla types and their topographical distributions implies fine-tuned functional specialisation associated with different feeding strategies and mechanical demands.

Taken together, these observations underscore the aphid labium as a multifunctional, evolvable structure integrating mechanical guidance, sensory feedback, and behavioural control. Its modular organisation allows repeated, independent evolutionary modifications that mirror ecological diversification and host-plant adaptation across the Aphididae.

This comparative analysis highlights the pivotal role of sensilla modification in aphid ecological diversification, supporting the hypothesis that the sensory evolution of the rostrum is closely tied to feeding strategy and host tissue type.

## 5. Conclusions

This comparative analysis highlights the aphid labium as a multifunctional organ integrating mechanical, sensory, and behavioural functions.

The apical sensory field of the last rostral segment shows a high degree of structural consistency across the studied aphid species, particularly in the number and arrangement of sensilla basiconica. Two main patterns were identified: concentric rings of eight pairs of Sb3 sensilla of varying lengths, and a configuration of seven pairs of Sb2 sensilla. Although variation exists in the types, length, and distribution of sensilla on the labial surface among species, it suggests a specific functional role.

The findings underscore that rostral segmentation, sensillar diversity, and cuticular specialisation jointly represent adaptive responses to the physical and structural diversity of host-plant tissues. Future research integrating ultrastructural imaging, neurophysiological assays, and behavioural analyses will be essential to clarify the specific mechanosensory roles of individual sensilla and to test hypotheses concerning the evolutionary trajectories of rostral morphology within the Aphididae.

## Figures and Tables

**Table 1:** Sensilla length statistics of aphid species (mean ± standard deviation; Unit: μm). No data means the species do not have this sensilla type. Sample size (St1–2, St4, Sb2–3 = 8; St5, Sb1, Sb4–5 = 5; St3 in *Trama* sp. = 5; St3 in other species = 8); St1–5: sensilla trichodea 1–5; Sb1–5: sensilla basiconica 1–5.

	St1	St2	St3	St4	St5	Sb1	Sb2	Sb3	Sb4	Sb5
*Uroleucon* sp.			46.9 ± 8.9			14.7 ± 2.0		4.6 ± 1.0		
*G. betulae*			45.3 ± 10.2			13.2 ± 1.4		3.6 ± 1.1		
*M. cerasi*			28.7 ± 9.7			10.5 ± 2.9		3.7 ± 0.5		
*P. juglandis*	74.8 ± 13.2		22.6 ± 5.8			11.2 ± 1.1		5.2 ± 0.8		
*Trama* sp.		80.6 ± 13.5	31.1 ± 1.9		47.9 ± 2.6	21.6 ± 1.0	11.1 ± 2.8			
*Chaitophorus* sp.	69.2 ± 18.8					12.9 ± 2.5		6.5 ± 1.0		
*L. roboris*		88.4 ± 23.7				20.8 ± 3.8	16.5 ± 3.7			
*Forda* sp.			27.1 ± 1.9			13.2 ± 2.5		4.7 ± 1.2	9.7 ± 1.0	9.2 ± 0.7
*P. cimiciformis*				39.0 ± 9.3		21.8 ± 5.8		6.8 ± 1.4		

**Table 4:** Diverse labial segments and mechanosensilla and feeding ecology.

Feeding Niche	Representative Species	Structural–Sensory Adaptations
Soft herbaceous shoots/leaves	*Uroleucon* sp., *Glyphina betulae*, *Myzus cerasi Chaitophorus* sp.	Short, 4-segmented labium; moderate or sparse ventral sensilla (*trichodea 1/3*, *basiconica 1/3*); banded spiniform tubercles in some. Optimised for gentle probing of thin epidermis.
Woody hosts (tree twigs)	*L. roboris*	Long, 5-segmented labium; abundant *trichodea 2*; sensilla on all surfaces; cuticular processes—enables deep penetration through lignified tissues.
Subterranean herb/root feeders	*Trama* sp., *Forda* sp.	5-segmented labium, dense sensilla arrays (*trichodea 2–5*, *basiconica 4–5*); cuticular processes for mechanosensory precision underground in Trama.
Root-associated, polymorphic species	*P. cimiciformis*	Long labium, sensilla numerous (*trichodea 4*); honeycomb surface texture (Figure 13C) may improve structural flexibility and sensory feedback.
Vein and midrib specialists	*P. juglandis*	Stout, short labium with dense *trichodea 1*; suited for firm tissues.

## Data Availability

The original contributions presented in the study are included in the article. Further inquiries can be directed to the corresponding author.
